# Understanding the Modus Operandi of MicroRNA Regulatory Clusters

**DOI:** 10.3390/cells8091103

**Published:** 2019-09-18

**Authors:** Arthur C. Oliveira, Luiz A. Bovolenta, Lucas Alves, Lucas Figueiredo, Amanda O. Ribeiro, Vinicius F. Campos, Ney Lemke, Danillo Pinhal

**Affiliations:** 1 Institute of Biosciences of Botucatu, Department of Genetics, Sao Paulo State University (UNESP), Botucatu, Sao Paulo 18618-689, Brazil; arthur.c.oliveira@unesp.br (A.C.O.); lucas.ac.silva@unesp.br (L.A.); dipietroneves@gmail.com (L.F.); ao.ribeiro@unesp.br (A.O.R.); 2 Department of Physics and Biophysics, Sao Paulo State University (UNESP), Botucatu, Sao Paulo 18618-689, Brazil; uiz.bovolenta@unesp.br (L.A.B.); ney.lemke@unesp.br (N.L.); 3 Laboratory of Structural Genomics (GenEstrut), Technology Developmental Center, Graduate Program of Biotechnology, Federal University of Pelotas, Pelotas, Rio Grande do Sul 96010-610, Brazil; fariascampos@gmail.com

**Keywords:** microRNA regulation, microRNA modules, mRNA fold-change, functional enrichment, gene regulatory networks

## Abstract

MicroRNAs (miRNAs) are non-coding RNAs that regulate a wide range of biological pathways by post-transcriptionally modulating gene expression levels. Given that even a single miRNA may simultaneously control several genes enrolled in multiple biological functions, one would expect that these tiny RNAs have the ability to properly sort among distinctive cellular processes to drive protein production. To test this hypothesis, we scrutinized previously published microarray datasets and clustered protein-coding gene expression profiles according to the intensity of fold-change levels caused by the exogenous transfection of 10 miRNAs (miR-1, miR-7, miR-9, miR-124, miR-128a, miR-132, miR-133a, miR-142, miR-148b, miR-181a) in a human cell line. Through an in silico functional enrichment analysis, we discovered non-randomic regulatory patterns, proper of each cluster identified. We demonstrated that miRNAs are capable of equivalently modulate the expression signatures of target genes in regulatory clusters according to the biological function they are assigned to. Moreover, target prediction analysis applied to ten vertebrate species, suggest that such miRNA regulatory modus operandi is evolutionarily conserved within vertebrates. Overall, we discovered a complex regulatory cluster-module strategy driven by miRNAs, which relies on the controlled intensity of the repression over distinct targets under specific biological contexts. Our discovery helps to clarify the mechanisms underlying the functional activity of miRNAs and makes it easier to take the fastest and most accurate path in the search for the functions of miRNAs in any distinct biological process of interest.

## 1. Introduction

About two decades ago, the discovery of a comprehensive variety of miRNAs—small endogenous non-coding RNAs—broadened horizons and revolutionized scientific knowledge regarding the mechanisms of regulation of gene expression in the metazoan genome. The ancestral origin of miRNAs, their dramatic expansion in bilateral animals, and their role in providing robustness to cellular transcriptional programs [1,2] suggest these molecules are protagonists in the evolution of functional complexity in living organisms [3,4].

MiRNAs have been reported to regulate a myriad of distinct biological processes, from cell differentiation and proliferation [5] to the onset of diseases such as cancer [6]. Mature miRNAs mainly act post-transcriptionally, modulating gene expression by pairing with complementary sequences preferentially at target 3’UTR region [7]. In animals, miRNAs first block the translation of target genes, which promotes the premature degradation of the mRNA, thus affecting final protein output [8].

Recently, advances both in experimental and computational methods have increased the availability of new genomes and transcriptomes thus providing resourceful data about sequence and expression levels of miRNAs and their respective target genes. However, while it is excessively laborious to query the wide bunch of biological functions of miRNAs and targets empirically through in vivo studies, computational strategies have afforded novel valuable insights on biological research as they paved the way for the validation of miRNA functions by yielding statistically significant hypotheses from the extensive collection of biological measurements [9].

Offshoots of these burgeoning datasets generated have shed light on diverse aspects of miRNA biology, including the (co)evolution of miRNAs and their target genes [10] and the existence of miRNA-target modules as part of extensive networks that control multiple cellular processes [11].

The peculiar miRNA-target pairing admits the occurrence of a broad spectrum of interactions, implying that a single miRNA can interact and regulate multiple genes, and/or a single gene can be regulated by several miRNAs. In fact, every miRNA may simultaneously control several target genes enrolled in a wide range of ongoing metabolic pathways within a single cell. This is relevant, as the unraveling of true interactions coupling miRNA to target genes to functions has demonstrated the existence of patterns or modules in post-transcriptional gene regulation mediated by miRNAs. Modules seem to evolve in a fashion that allows miRNAs to coordinately exert cooperative effects on functionally related target genes [12]. Currently, however, it is not clear how miRNAs are able to independently and accurately regulate levels of each of their targeted mRNAs, providing specific transcript outputs required by multiple concurrent biological processes.

Several studies have sought to describe features involved in the miRNA-target recognition, such as seed match [7] and site accessibility [13], and how these variables affect the intensity of the regulation mediated by a miRNA. However, these studies have only demonstrated variations between miRNA-target interactions individually, apart from the biological context they are assigned to. Other studies have demonstrated that cellular localization of the miRNAs and their targets, and the competition between the target sites available in the transcriptome are important features that drive the gene regulation provided by the miRNAs in a broader cellular context [14], shedding new insights on how miRNAs coordinate gene expression post-transcriptionally.

In this paper, we seek to test the hypothesis of whether miRNAs regulate with similar intensities the expression profiles of genes enrolled in closely related biological functions. Using an in-depth detailed analysis of a broad set of global expression data of target messenger RNAs after perturbation of the expression of 10 selected miRNAs, we characterized the differential level of regulation/modulation of targets expression profiles and its potential relationship to the different functions exerted by miRNAs. We discovered a complex and highly organized scheme of gene expression levels control, which depends on the intensity of repression of the miRNA over target genes and it is structured into interdependent regulatory cluster. Our analysis shows that regulatory clusters are not limited to miRNA canonical targets, but also rely on other types of miRNA regulation which comprises a significant part of the miRNA regulatory networks, thereby clarifying the mechanisms underlying the functional activity of different miRNAs and facilitating a more meaningful analysis of their enrichment into distinct biological processes.

## 2. Results

### 2.1. Functional Enrichment Terms Distribution Along miRNA-Responsive Gene Clusters

By analyzing gene expression alterations following the transfection of 10 miRNAs in HeLa cells (data obtained from Garcia et al. [15]), we found that the transcript set in a given biological system can be grouped into regulatory clusters according to the degree of responsiveness to the introduced miRNA, measured by absolute messenger RNAs (mRNAs) fold changes. We identified an average of 4.7 clusters of mRNA fold changes per miRNA: the transfection of miR-1, miR-7, miR-124, and miR-142 resulted in four clusters each, while miR-9, miR-128, miR-132, miR-133a, and miR-181a transfection returned five clusters and miR-148b returned six clusters (Supplementary Figure S2). We identified three types of clusters: (I) up-regulated genes, (II) down-regulated genes, and (III) genes tended to do not be regulated by the transfected miRNA. In the first two types of clusters, all genes were either up or down-regulated, while the last cluster comprises a group of genes with little fold change variation (neither up- nor down-regulated) and genes with no variation at all (zero fold change).

**Figure 1:**
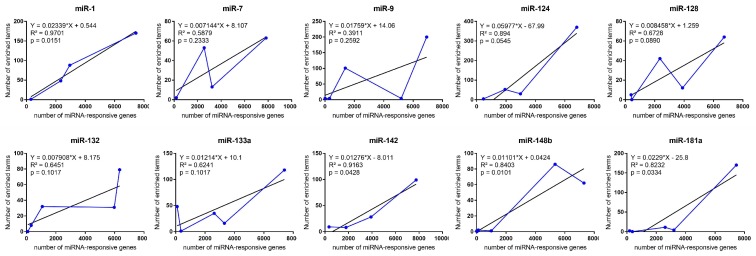
Correlation between the number of enriched terms and the number of miRNA-responsive genes in each cluster. Clusters are ordered according to the number of gene members embraced.

After, we performed functional enrichment analysis on each regulatory cluster in order to identify putative regulatory properties of such clusters. Interestingly, we observed that for six out of the 10 analyzed miRNAs (miR-7, miR-9, miR-124, miR-128a, miR-132, and miR-133a), there was no correlation between the number of enriched terms and the number of the mRNAs within each cluster (*p*-value < 0.05; Figure 1). See Supplementary Table S1 for a detailed list containing the number of enriched terms and mRNAs inside each regulatory cluster.

To verify whether the observed term-enrichment distributions (i.e., number of terms and the correlation between number of terms and number of mRNA in each cluster) is randomly distributed or not, we performed an identical enrichment analysis on random datasets constituted of artificial clusters containing the same number of genes of each analyzed cluster, and tested their distribution through a chi-squared test (Supplementary Table S2). Only seven out of the 47 verified clusters (one from miR-1; one from miR-132; one for miR-133a; three from miR-148b; and one from miR-181a) sustained the H0 hypothesis (i.e., random distribution of the enriched terms), reinforcing the idea that the observed term-enrichment distributions are not random but rather resulted from specific miRNA control. In fact, the random cluster sets showed high correlations between the number of responsive miRNA-genes and the number of enriched terms in each cluster as expected (R^2^ > 0.8; Supplementary Figure S3), while the opposite was observed for the inferred non-random gene clusters. Moreover, most clusters showed higher numbers of enriched terms in the studied groups than in the random sets, suggesting higher aggregation of genes related to common biological processes in the studied group than it would be expected by chance (Supplementary Table S2).

**Figure 2:**
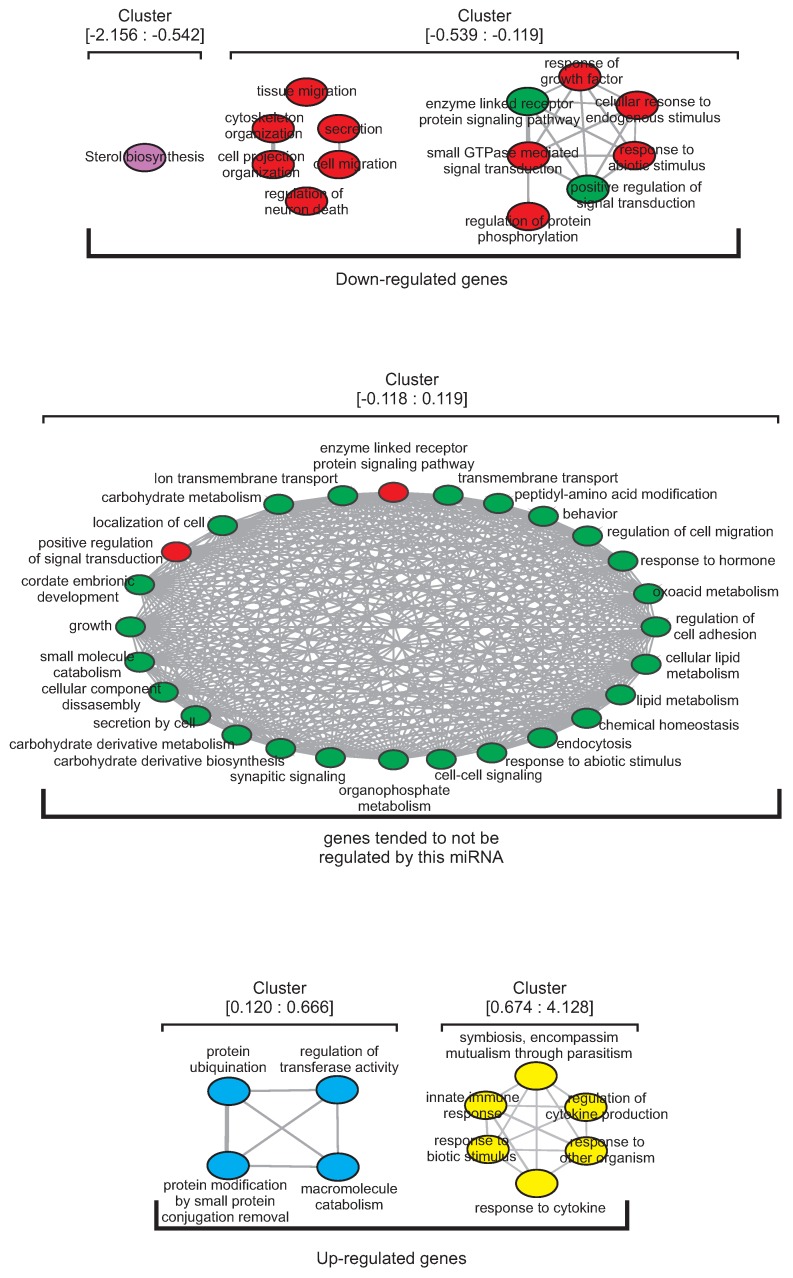
Functional enrichment of messenger RNA (mRNA) clusters after miR-133a transfection. Purple terms belongs to the cluster (−2.156:−0.542), red terms to the cluster (−0.539:−0.119), green terms to the cluster (−0.118:0.119), blue terms to the cluster (0.120:0.666) and yellow terms to the cluster (0.674:4.128). The numbers within brackets represent the minimum and maximum fold change values of each cluster. Terms with different colors inside a cluster indicate that the same term is also present in the cluster of that color. Grey edges connect highly similar terms, and the edge width indicates the similarity degree. Results for other miRNAs can be visualized at Supplementary Figure S4 and a detailed description of the terms found in each cluster can be visualized at Supplementary Table S3.

Along with the observed differential quantitative distribution of enriched terms among clusters, we proposed two possibilities for what we would expect from a distinctive qualitative distribution: (i) several enriched terms would take part in more than one cluster, or (ii) enriched terms would tend to be present only in a unique cluster. Interestingly, by comparing the functional enriched terms within each cluster, we observed almost no overlap between clusters (Figure 2; Supplementary Figure S3; Supplementary Table S3).

As shown in Figure 2 (and in more details in Supplementary Table S3), for miR-133a we observed that only two enriched terms overlap between cluster (−0.118:0.119) and cluster (−0.539:−0.119): “Positive regulation of signal transduction” and “Enzyme linked receptor protein signaling pathway”. Strikingly, in each cluster almost all terms were connected as semantically related by REVIGO, suggesting that not only genes contained in one regulatory cluster have different biological functions in comparison to genes contained in other regulatory clusters from the same miRNA, but also genes within the same regulatory cluster tend to be engaged in similar functions. For instance, clusters (−2.156:−0.542) and (−0.539:−0.119) represent the down-regulated genes after miR-133a transfection. The former has only one enriched term (Sterol biosynthesis), suggesting that genes in this cluster may be associated with this specific biological function. On the other hand, the latter is the cluster with the highest number of different functions (i.e., without connections between them). The greatest subcluster groups terms related to “response to signal stimulus” and “signal transduction regulation”. Additionally, terms related to “cell secretion and organization”, “tissue migration” and “regulation of neuron death” are found at this cluster. Interestingly, these terms, although not semantically connected, are not completely unrelated, potentially being all associated with a greater function of neuronal organization regulation and signaling functions. Cluster (−0.118:0.119), which contains enriched biological functions apparently not regulated by miR-133a, encompass enriched terms semantically related to each other. Overall, terms were mainly associated with cellular compound metabolism, like “carbohydrate metabolism”, “lipid metabolism”, “organophosphate metabolism” and “oxoacid metabolism”, suggesting that miR-133a may not be associated with the regulation of metabolism-related genes. The clusters (0.120:0.666) and (0.674:4.128) represent the up-regulated genes after miR-133a transfection. Cluster (0.120:0.666) contains four semantically related enriched terms: “protein ubiquitination”, “protein modification by small protein conjugation or removal”, “macromolecule catabolism”, and “regulation of transferase activity”, suggesting a role for this cluster in the coordination of protein maturation. Finally, cluster (0.674:4.128) comprehends five terms, also semantically related: “symbiosis, encompassing mutualism through parasitism”; “response to other organism”; “innate immune response”; “response to cytokine production" and “response to biotic stimulus”. All these terms can be related to immune system responses to an invasive organism, such as a virus or bacteria, suggesting a role for (0.674:4.128) cluster in the regulation of such protective function. These data support the idea that miRNAs act through regulatory clusters, distinctively modulating mRNA levels (i.e., the degree of the mRNA fold change) depending on the biological function the targeted mRNAs are enrolled with. Moreover, shows that miRNAs regulate functionally related genes with similar intensity, which reflects a more parsimonious strategy to cope with the control of genes associated with the same biological function.

**Figure 3:**
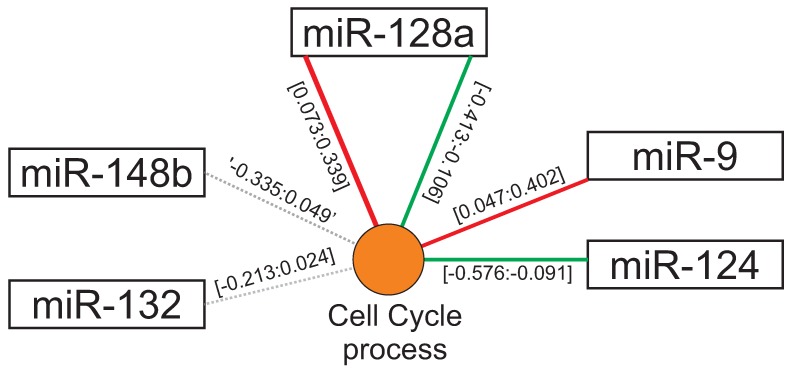
Combined action of distinct regulatory clusters. Each line represents a regulatory cluster connecting miRNAs to the enriched biological term associated with the cluster. The numbers within brackets represent the minimum and maximum fold change values of that cluster. Red lines represent up-regulated gene clusters, green lines represent down-regulated gene clusters, and gray dashed lines represent clusters of genes apparently not regulated by the miRNA.

Regulatory clusters from distinct miRNAs can also be enrolled in correlated biological functions. For instance, miR-9 cluster (0.047:0.402), miR-124 cluster (−0.576:−0.091), miR-128a clusters (−0.413:−0.106) and (0.073:0.339) control genes related to “cell cycle process” (Figure 3). These results suggest that regulatory clusters from different miRNAs may act in combination and confer a more precise regulation of a particular biological function. The “cell cycle process” was also present in the miR-132 cluster (−0.213:0.024) and miR-148b cluster (−0.335:0.049), suggesting that these miRNAs tend to do not interfere in cell cycle events. Interestingly, miR-128a has two clusters with antagonistic effects on this biological function (cluster (−0.413:−0.106) and cluster (0.073:0.339)) suggesting that in some cases clusters of genes targeted by the same miRNA may also cooperate to each other into regulatory networks.

### 2.2. Protein-Coding Genes with Present/Absent Predicted miRNA Target Sites Inside miRNA-Responsive Gene Clusters

**Figure 4:**
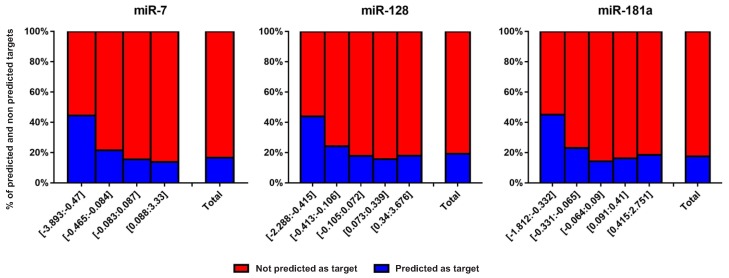
Proportion of genes predicted and do not predicted as targeted by miRNAs belonging to each cluster of miRNA-responsive genes for miR-7, miR-128, and miR-181a. Additional data of other miRNAs can be found in the Supplementary Figure S5.

To access how many of the miRNA-responsive genes were, in fact, interacting with the miRNAs, we performed a target prediction analysis using TargetScan 7 [16] target prediction tool. Interestingly, we observed that only a small fraction of the perturbed genes was predicted to be under direct interaction with the miRNAs (Figure 4; Supplementary Figure S5). This data suggest that a significant part of mRNA fold changes came from non-canonical miRNA regulation. In other words, a silencing-independent miRNA regulation, or a direct action of a given miRNA over one or some external mRNAs occurs and leads to a cascade effect, promoting indirect regulation of several other genes of a given biological pathway.

It is widely known that target prediction tools still return a considerable number of false negative results. To address this issue, we accounted all targets predicted by TargetScan (not only the conserved ones), in order to maximize sensitivity values and obtain a better estimation regarding the proportion of genes with or without target sites among the responsive genes. However, to access if we were still missing a significant amount of data, we searched for experimentally validated targets of the 10 miRNAs to evaluate how many of them were not predicted by TargetScan. We identified 699 validated targets, of which 354 (an average of 35.4 targets per miRNA) had not been predicted as targets by TargetScan (see Supplementary Tables S4 and S5 for a detailed list of the targets experimentally validated). In this sense, we can expect that there are other canonical interactions that were not identified in our analysis. Nevertheless, even considering this limitation, there would still be an expressive number of non-canonical interactions performed by miRNAs, highlighting the impact of miRNAs in a broader context. Future updates of the target prediction tools providing improved sensibility values are welcome towards a better estimation of the proportion of the miRNA canonical regulation.

For all miRNAs here analyzed, clusters containing down-regulated genes had higher proportions of predicted canonical interactions in relation to the whole set of perturbed genes. As expected, clusters encompassing genes apparently not regulated by the miRNAs and up-regulated genes showed a smaller proportion of predicted canonical interactions compared to the down-regulated gene clusters. The only exception was miR-148b, whose cluster (−6.795:−6.473) showed a smaller proportion of predicted targets than cluster (4.252:6.957). However, this may be an artifact caused by the small number of perturbed genes (only seven) in the (−6.795:−6.473) cluster. Here, we describe canonical interactions as the silencing properties of miRNAs over their target genes.

**Figure 5:**
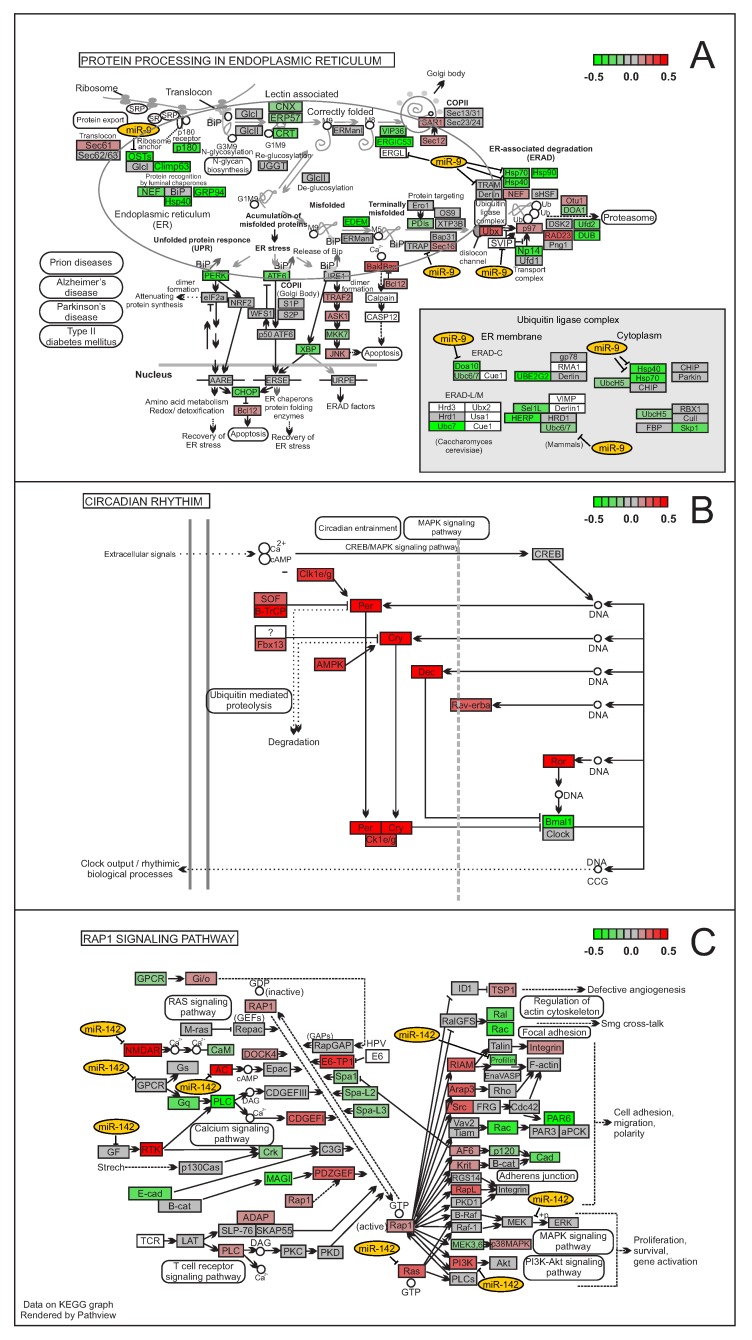
miRNA-responsive biological pathways and the presence or absence of interactions between its genes and the transfected miRNA. (**A**) predicted interactions among miR-9 (yellow ellipses) and the mRNAs of a biological pathway perturbed after miR-9 transfection; (**B**) miR-1 responsive biological pathway with no predicted interaction between miR-1 and its genes; (**C**) predicted interactions among miR-142 (yellow ellipses) and genes either up- or down-regulated after miR-142 transfection. Green rectangles represent down-regulated mRNAs. Red rectangles represent up-regulated mRNAs. Gray rectangles represent mRNAs with little-to-no fold change after miRNA transfection.

Additionally, even though most of the enriched biological pathways contain genes predicted as canonical targets of the analyzed miRNA (Figure 5A), some biological pathways responsive to miRNA transfection do not have any interaction predicted by TargetScan in relation to up- or down-regulated mRNAs (6 out of 75 analyzed; Figure 5B), suggesting that some miRNA-mediated functions could be entirely controlled by non-canonical regulation. Interestingly, in some biological pathways, miRNAs that were predicted as targets by TargetScan were also up-regulated after miRNA transfection (Figure 5C).

### 2.3. Conservation of miRNA Regulatory Clusters in Vertebrates

**Figure 6:**
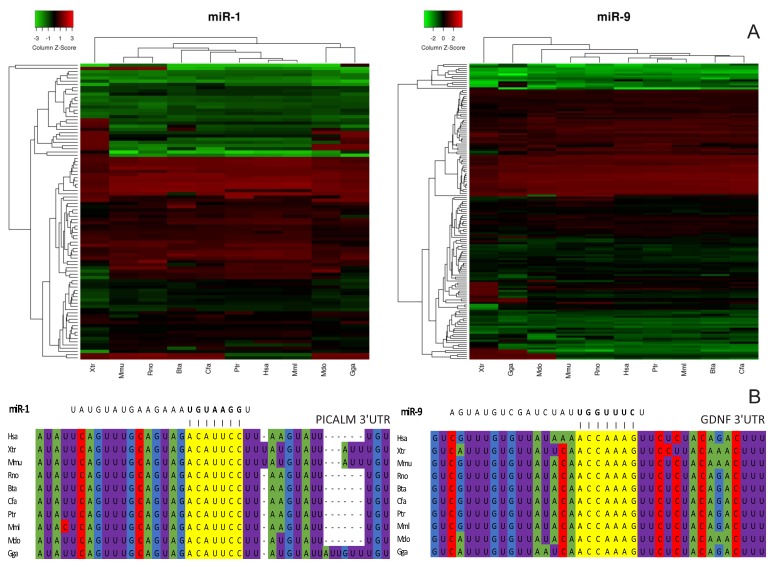
Conservation of target responsiveness over miRNA interaction. (**A**). Heatmaps of Context++Scores for miR-1 and miR-9 predicted targets among 10 vertebrate species. Negative Context++Score values are represented in green, positive values are represented in red, and near zero values are represented in black. Lower Context++Score values represent stronger miRNA regulation levels. Heatmaps for other miRNAs can be found in the Supplementary Figure S7. (**B**). Example of evolutionary conservation of the 3’UTR of two genes targeted by miR-1 (PICALM) or miR-9 (GDNF) in the genome of the ten vertebrate species. Yellow marks represent the miRNA binding site at the target mRNA 3’UTR.

To verify whether this regulatory mechanism is evolutionarily conserved in the vertebrate tree of life, we performed a target prediction analysis on 10 species (*Homo sapiens*, *Pan troglodytes*, *Macaca mullata*, *Mus musculus*, *Rattus norvegicus*, *Bos taurus*, *Canis familiaris*, *Monodelphis domestica*, *Gallus gallus*, and *Xenopus tropicalis*) using TargetScan7 and compared the Context++Score obtained for each interaction. We found the Context++Score has shown a high correlation to the fold change of the target mRNA after miRNA transfection (R^2^ = 0.9736; Supplementary Figure S6). Most targets of a given miRNA have similar Context++Scores for all vertebrates analyzed (and thus similar fold changes after miRNA transfection; Figure 6; Supplementary Figure S7), suggesting this intensity-based mechanism of target expression levels modulation mediated by miRNAs is a conserved functional feature encoded in the vertebrate genomes.

We also observed that phylogenetically related groups presented more similar Context++Scores for a given miRNA and its targets compared to more distant groups. For instance, miR-1 target regulation in primates (*H. sapiens*, *P. troglodytes* and *M. mullata*) and rodents (*M. musculus* and *R. norvegicus*) exhibited a closer pattern within these groups than to other species. Additionally, placental mammals (*H. sapiens*, *P. troglodytes*, *M. mulata*, *M. musculus*, *R. norvegicus*, *B. taurus* and *C. familiaris*) showed more similar Contex+t+Scores among each other than in relation to the external group (*M. domestica*, *G. gallus* and *X. tropicalis*). In the same way, Context++Scores for miR-9 and its targets are closer among mammals, slightly increasing the divergence with phylogenetic distance.

## 3. Discussion

### 3.1. MiRNAs Regulatory Clusters Impact on Biological Functions

In this work, we gather evidence that miRNAs can individually coordinate the regulation of a broad range of distinct genes by inducing similar fold-changes on those with related biological functions. This creates miRNA regulatory clusters that control similar functions with similar pairing intensities and distinct functions with distinct potentiality, allowing each independently regulated cluster interact with other clusters from distinct regulatory networks without interfering in the stability of each other. Interestingly, we found that most enriched functional terms within each miRNA regulatory cluster are functionally related. This connection of terms supports not only the idea of an aggregation of genes with identical functions but also match to the hypothesis of the presence of related biological roles on single regulatory clusters.

Like miRNA modular regulation [11,17], miRNA regulatory clusters are highly dynamic, exhibit variation regarding mRNA fold change levels and the number of gene members within each cluster, as well as in relation to the number of clusters itself. As the microarray datasets we analyzed contained miRNA transfections only in HeLa cells, we cannot draw further inferences on the dynamics of miRNA clusters on different cell lines. Since miRNA functions are highly dependent on the collection of protein-coding genes expressed in the cell [18], we expect that the properties of each cluster (e.g., biological function assignment and the number of mRNAs in a cluster) would differ depending on cells environment. Thus, additional experiments involving miRNA transfection inside different cell lines would be helpful to better elucidate the behavior of regulatory clusters in different scenarios.

Regulatory clusters of distinct miRNAs can cooperate to control genes involved in similar biological functions, as shown in Figure 3. In such cases, the combined regulatory intensities provided by each miRNA cluster can enhance the usual fine-tune regulation of gene transcripts. This helps to explain how a given miRNA can concurrently participate in several regulatory modules and how the same miRNA module can be involved in several biological functions [19]. Under this context, miRNA modules are founded by the aggregation of different miRNA regulatory clusters.

The clusterization of miRNA targets according to their biological functions appears to be a conserved feature of vertebrates’ genomes. This association can be, at least, partially explained by miRNA pairing properties, since such properties are associated with the miRNA potentiality of regulating its target [20]. MiRNA regulatory elements (MREs) (i.e., the miRNA site recognition region present on the target) are highly conserved throughout vertebrates [21,22], thus being able to contribute to the maintenance of miRNA regulatory clusters throughout vertebrate’s evolution. However, despite of the great association between Context++Scores and miRNA down-regulation, cell environment changes from species to species thus requiring further experiments involving miRNA transfection into distinct cell lines from multiple species to confirm such conserved pattern.

The identification of distinct miRNA regulatory clusters also raised the question whether other non-coding RNAs (ncRNAs), such as PIWI-interacting RNAs (piRNAs), tRNA-Derived Fragments (tRFs), small nuclear RNAs (snRNAs) and small nucleolar RNAs (snoRNAs) would act in a similar manner or not. However, for some ncRNAs such as piRNAs, this becomes difficult to associate, because the rules governing piRNAs target recognition are yet to be fully understood. It is known that piRNAs possibly do not have recognition elements in their targets as do have miRNAs, although some useful nucleotide signatures for its identification exist (such as an Adenine in position 10 and a Uracil in position 1) [23]. However, other ncRNAs such as tRFs may act in a similar manner as miRNAs in specific cases, mostly in stress response [24]. In this sense, future studies focused on other ncRNAs would help to better address molecular rules driving interactions and the control of target genes expression.

### 3.2. Beyond miRNA Pairing: Regulation of Non-Canonical Targets

Even though most of the knowledge obtained regarding miRNA regulation comes from the study of canonical interactions, we demonstrate that they comprehend only a fraction of the miRNA regulatory universe, indicating that miRNAs may undertake a larger and more complex regulatory mechanism than simply interact with its target genes to promote mRNA/protein down-regulation. Although we have sought to reduce methodological bias by accounting all miRNA targets predicted by TargetScan (and not only the conserved ones), miRNA target prediction still lacks in sensitivity, often returning false-negative results. Our analysis demonstrated that approximately 51% of the validated targets of the analyzed miRNAs were not predicted by TargetScan. However, this number represents only a small fraction of the total predicted targets (10,858 predicted targets versus 354 validated targets not predicted by TargetScan). It is important to keep in mind that the yet limited number of validated targets available in the literature (699 for the 10 miRNAs analyzed) makes it very difficult to estimate the real proportion of missing interactions. The main reason for that is the possible bias when choosing candidate targets to validate. Once the target prediction tools available contain distinct parameters, they identify distinct sets of predicted targets. This creates a scenario in which target prediction tools tend to identify more or less validated targets depending on the frequency and purpose of its use (e.g.,: identify targets and validate them experimentally or to perform bioinformatic analysis with large scale data). In this sense, the validation of an increasing number of new interactions as well as new updates of the target prediction tools will allow a more detailed analysis of the real proportion of canonical and non-canonical regulation done by miRNAs.

As expected, we observed that the relative number of non-canonical targets was inversely related to the potency of target gene down-regulation. Clusters in which down-regulation was stronger had more interactions predicted by TargetScan than clusters with weak down-regulatory efficiency or even up-regulated genes. However, even within the clusters with stronger down-regulation, about half of the perturbed genes were not predicted by TargetScan as targets.

Several studies have highlighted atypical mechanisms in which miRNAs may perform functional regulation [25]. For example, miRNAs were identified as capable to activate toll-like receptors, promoting inflammatory responses in lung cancer by miR-21 and miR-29a [26] and neurodegeneration by let-7 [27] activations of TLR7. Additionally, Eiring et al. [28], described a hybridization of miR-328 with the non-Ago protein hnRNP E2, which releases CEBPA from translation inhibition caused by hnRNP E2. Other reports demonstrated an interaction of miR-181c with mitochondrial transcripts [29], even that mitochondria do not encode miRNAs themselves. In addition to these non-canonical interactions, miRNA often target several transcription factors, suppressors and other regulatory genes that potentially interfere in a cascade of genes and biological pathways, being able to promote the regulation of downstream targets.

The similar number of down- and up-regulated genes identified after miRNA transfection also suggest a massive role of miRNAs as gene enhancers. Previous studies have described the ability of miRNAs to up-regulate protein output post-transcriptionally [30,31,32] by either the direct activation of the mRNA target or repression attenuation. In addition to the up-regulation on protein expression, in some cases, mature miRNAs can be imported back to the nucleus to induce gene transcription [33]. Huang et al. [34] demonstrated that miR-744 and miR-1186 can induce transcription activation of Ccnb1 by targeting its promoter, in murine cells. Also, miR-483-5p, located in an intron of IGF2, can enhance the expression of its host gene [35]. Likewise, lin-4 [36] and let-7 [37], in *C. elegans*, can recruit other proteins to promote autoactivation and final maturation of its own precursors, respectively.

**Figure 7:**
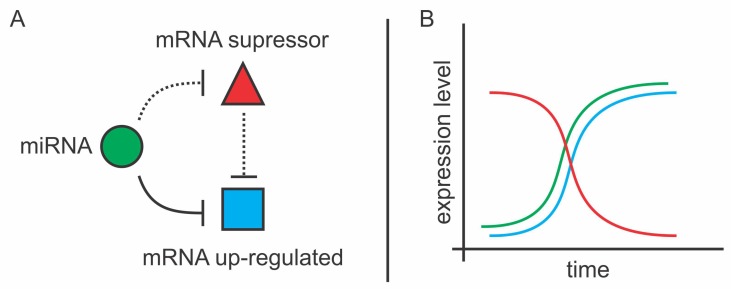
MiRNA-mediated mRNA up-regulation scheme. (**A**) Interaction network between the miRNA, the up-regulated mRNA, and its suppressor. The miRNA down-regulates mRNA suppressor while fine-tunes the expression of the up-regulated mRNA. The continuous line represents direct interaction and dashed line represents either direct or indirect regulation; (**B**) Representative expression variation of the network members. Green line represents miRNA expression, the blue line represents up-regulated mRNA expression and the red line represents mRNA suppressor expression.

Interestingly, some up-regulated mRNAs were also predicted as bonafide targets of the 10 transfected miRNAs. In this case, it is likely that the miRNA promotes up-regulation of that gene (either by direct or indirect regulation as described above) and also directly modulates its protein output by fine-tuning its expression (Figure 7) [38].

Altogether, such miscellaneous of silencing-independent properties of miRNAs could help explain the massive number of observed mRNA fold-changes without a direct interaction predicted by TargetScan. Future studies focusing on these unusual mechanisms, which still stand for a very limited number of reported cases, may help to better address miRNA functioning under distinct biological contexts. Currently, we still lack predictive tools and computational models to evaluate the impact of these regulations on a large scale.

### 3.3. How Do miRNAs Perform Cluster Regulation?

The realization that miRNAs are able to coordinate the regulation of highly distinct biological functions by sorting them into regulatory clusters raises the question of how miRNAs perform such clusterization. Although our analysis per se may not be sufficient to solve that question in full, the vast knowledge regarding miRNA properties allows us to draw hypothesis on how they could orchestrate such molecular mechanism.

An important feature of miRNA regulatory dynamics is the occurrence of competition between all possible sites that can be targeted by a single miRNA. Usually, in a cell, the total number of available sites, accounted by the number of sites in all transcripts times the expression of each of them overpass the number of miRNAs available [14,39], leading to a competition of those sites for the same limited miRNA. In this sense, target genes with high-affinity binding sites are more sensitive to miRNA regulation than those with low-affinity [39,40]. Additionally, the cellular environment is very complex and due to variations in miRNA local concentrations and spatial co-localization, there is a considerable difference between the total number of molecules of a given miRNA in the cell and the number that is effectively available to interact with each target site [14]. MiRNAs and their targets have been identified in several subcellular compartments, such as rough endoplasmic reticulum [41], P-bodies [42], lysosomes [43], mitochondria [44], nucleus [45] and others. The association of divergent local concentrations of miRNAs and their targets, with the distinct affinities of the target sites in each microenvironment, could potentially help to promote the formation of the regulatory clusters. A novel technology of subcellular RNA-seq [46] able to quantify miRNA and protein-coding transcripts in different microenvironments would help to further elucidate this scenario.

### 3.4. Future Perspectives

Our results disclose the underlying mechanism by which miRNAs accurately regulate cell transcriptome, opening new doors to the miRNA research field and raising several new questions that can lead to interesting forthcoming investigations.

For example, one of the greatest limitations of using miRNAs as therapeutic targets is the high number of off-targets [47] and undesirable side effects [48] of their introduction, even leading to cell death in drastic cases [49]. It is likely that non-canonical interactions are partially responsible for those effects. Thus, knowing the group of genes enrolled in the regulatory clusters of a miRNA and the biological processes they rule out will provide a better estimate of the main functions that would be perturbed and their putative severity after the manipulation of that specific miRNA. Moreover, the study of the regulatory clusters in a broader range of cellular contexts can potentialize the use of miRNAs as biomarkers, by providing a better view of the biological pathways and processes that are unregulated with the abnormal expression of each miRNA. In both cases, the better understanding of such interactions, as provided in the present paper, could help on transforming miRNAs in efficient therapeutic agents.

Additionally, we list here some other researches that would benefit from the knowledge of miRNA regulatory clusters:

- MiRNAs shares similar regulatory properties with several non-coding RNAs (ncRNAs), such as tRFs and other siRNAs. Studies focusing on identifying if regulatory clusters also occurs on other ncRNAs regulation could bring new insights on regulatory properties of non-coding RNAs.
- Target prediction analysis suggests that the regulatory clusters are a conserved phenomenon. This allows in vivo experiments to be performed in model organisms, bringing novel possibilities for studying how regulatory clusters would respond in systemic environments.
- Features of target binding sites may be largely relevant to the inception of regulatory clusters. However, our data shows that non-canonical and indirect interactions are part of miRNA regulation. Prospective high-throughput studies would benefit from this knowledge while performing functional enrichment analysis to map biological functions controlled by miRNAs.

## 4. Materials and Methods

### 4.1. Gene Expression Datasets Compilation

We analyzed 10 previously published microarray datasets that evaluated mRNA expression alteration after the transient transfection of distinct miRNA mimics into HeLa cells: miR-7 (GSM210897), miR-9 (GSM210898), miR-128a (GSM210903), miR-132 (GSM210904), miR-133a (GSM210907), miR-142 (GSM210909), miR-148b (GSM210911), miR-181a (GSM210913), miR-1 (GSM37599), and miR-124 (GSM37601) [15]. Since all these datasets were obtained from the same Agilent array platform, with a high confident miRNA-based repression signal and were pre-processed and normalized [16], non-specific effects and methodological bias should be considered irrelevant.

### 4.2. mRNA Fold-Changes Clustering

For each array, we clustered the mRNAs according to their fold changes ($log_{2}$) after miRNA transfection using the software Weka (v3.8; [50]) by the k-means method. Nine clustering formats were tested (from 2 to 10 groups) and the best cluster number for each array was identified by the elbow curve method (Supplementary Figure S2), using a python algorithm developed by Sotopaa et al. [51]. In each array, we identified three types of clusters: (I) up-regulated genes, (II) down-regulated genes, and (III) genes apparently not regulated by the analyzed miRNA (i.e., genes with little fold change variation [either up- or down-regulated] and genes with no variation at all [zero fold change]).

### 4.3. Functional Enrichment of mRNA Clusters

We performed functional enrichment analysis on each cluster using the ToppCluster tool (https://toppcluster.cchmc.org/) [52], searching for enriched Gene Ontology (GO) Biological Processes (BP) and Biological Pathways (KEGG) terms. ToppCluster tool allows the functional enrichment of several clusters simultaneously, returning an integrated network with all possible interactions and overlaps between them. Thus, it allows the evaluation of the degree of similarity between the clusters within each array. The enriched BP terms of each cluster were submitted to a semantics redundancy analysis by the REVIGO tool (http://revigo.irb.hr/; allowed similarity = 0.5) [53] in order to reduce semantic similar terms within each cluster. For the remaining terms, highly similar terms were linked by edges with the line width indicating the degree of similarity [53]. Finally, the generated networks were rendered in Cytoscape (v3.7.0) for better visualization [54].

### 4.4. Generation of Random Sets and Chi-Squared ($X^{2}$) Test

To generate the random sets we used all genes, regardless of their fold change. We randomly grouped the same number of genes identified in each cluster for each miRNA and, then, we performed the functional enrichment analysis of these random sets, using the same parameters used in item 4.3. Finally, we performed the chi-squared test to assess if the distribution of the enriched terms of the random sets (the observed values) matches the distribution of the original enriched terms identified (the expected values). For the chi-squared tests, we did not use REVIGO summarizing for either observed or expected values. All analyses were conducted in triplicates for each cluster.

### 4.5. Linear Regression Analysis

To identify the correlation between the number of enriched terms and the number of mRNAs contained in each cluster, we performed a linear regression analysis using GraphPad Prims software (v7.0; https://www.osbsoftware.com.br/produto/graphpad-prism/). For the random sets, the average number of terms of the triplicates were used.

### 4.6. MiRNA Target Prediction

MiRNA target prediction analysis was used to (i) evaluate the proportion of canonical and non-canonical interactions of the miRNAs analyzed, in both global context and within enriched biological pathways (Material and Methods Section 4.7 and Section 4.8, respectively) and (ii) to perform the evolutionary conservation analysis (Material and Methods Section 4.9).

For the evaluation of the proportion of canonical and non-canonical interactions, we obtained the complete miRNA target prediction data for the 10 miRNAs studied from the human (*Homo sapiens*) dataset of the TargetsScan website (v7.2; http://targetscan.org) [16], irrespective of site conservation. We did not limit our analysis to conserved targets in order to avoid underestimating the number of predicted targets in relation to the total number of perturbed genes since target prediction tools still lack in sensibility.

For the evolutionary conservation analysis we obtained TargetScan data for conserved sites from 10 vertebrate species (*Homo sapiens*, *Pan troglodytes*, *Macaca mullata*, *Mus musculus*, *Rattus norvegicus*, *Bos taurus*, *Canis familiaris*, *Monodelphis domestica*, *Gallus gallus* and *Xenopus tropicalis*).

### 4.7. Evaluation of the Proportion of Predicted and Not Predicted mRNAs

We evaluated the proportion between the total number of the responsive mRNAs and the number of miRNA-responsive targets that were predicted by TargetScan, for each cluster individually and for all clusters combined for each miRNA to access how many responsive genes were predicted to be under canonical regulation of the miRNAs.

### 4.8. Investigation of Validated miRNA Targets

To access how many targets predicted by TargetScan were previously validated, and how many validated targets of the miRNAs analyzed were not predicted by TargetScan, we used TarBase (v8; http://carolina.imis.athena-innovation.gr/diana_tools/web/index.php?r=tarbasev8%2Findex) [55] and miRTarBase (v7; http://mirtarbase.mbc.nctu.edu.tw/php/index.php) [56] databases. We selected these databases for their update policy (i.e., data is updated when new interactions are validated) and their collection and curation of data ([57]). However, even the best databases still lack great reliability, due to the scarcity of information in the literature and few multi-study validations of single interactions [57]. In this sense, we only selected highly supported interactions. For TarBase, we filtered the results by low-throughput, positive, and direct interactions, while for miRTarBase we only selected strong evidence validations (Reporter assay, western blot, and qPCR).

### 4.9. Analysis of Enriched Biological Pathways

We used the Pathview package (v.1.22.1) [58] from R Bioconductor (v.3.8) [59] on the “default” option to render the mRNA fold changes and map them into the enriched biological pathways identified by ToppCluster in each cluster. Additionally, Pathview was used to identify genes within each enriched pathway that was predicted by TargetScan to be targeted by a miRNA. Finally, both data were manually merged to generate a unique graphic representation.

### 4.10. Evolutionary Conservation Analysis

We compared the TargetScan Context++Score of the miRNA-target interactions for 10 vertebrate species to evaluate if the degree of responsiveness of mRNA targets, inferred through mRNA fold changes after miRNA transfections, was conserved across vertebrates. In order to improve data visualization and comprehension, we only consider target sites that were predicted into all 10 selected species, with exception of miR-132 and miR-142 that did not have any prior predicted target for *P. troglodytes* and *G. gallus* (miR-132), and *C. familiaris* (miR-142). Thus, such species were not integrated into the analysis of these miRNAs.

## 5. Conclusions

Our study reveals that miRNAs are able to simultaneously coordinate a wide range of biological functions by inducing similar fold changes in functionally related protein-coding genes, thereby aggregating them into specific regulatory clusters. Peculiarly, miRNA regulatory clusters are not limited to canonical targets, but also rely on non-canonical regulation which comprises a significant part of the miRNA regulatory universe. Our data also evidenced that this miRNA modus operandi is evolutionarily conserved implying that it must have been evolving adaptively in vertebrates. We envision that further experimental studies based on miRNA transfections in different cell lines and from several species will further help to pave the way for an even better picture of the modus operandi and evolution of miRNA regulatory clusters.

## Supplementary Materials

Supplementary materials can be found at https://www.mdpi.com/2073-4409/8/9/1103/s1.

## Abbreviations

The following abbreviations are used in this manuscript:

BP	Biological Process
GO	Gene Ontology
mRNA	messenger RNA
miRNA	microRNA
