# Supplementary material for: Understanding the Modus Operandi of MicroRNA Regulatory Clusters
Source: Cells. 2019 Sep 18;8(9):1103. doi: 10.3390/cells8091103 (PMC6770051; doi:10.3390/cells8091103)
Supplement: Supplementary file 1 [file cells-08-01103-s001.zip › Supplementary Figures.pdf]

**Supplementary Figure S1.** Summary of the workflow.

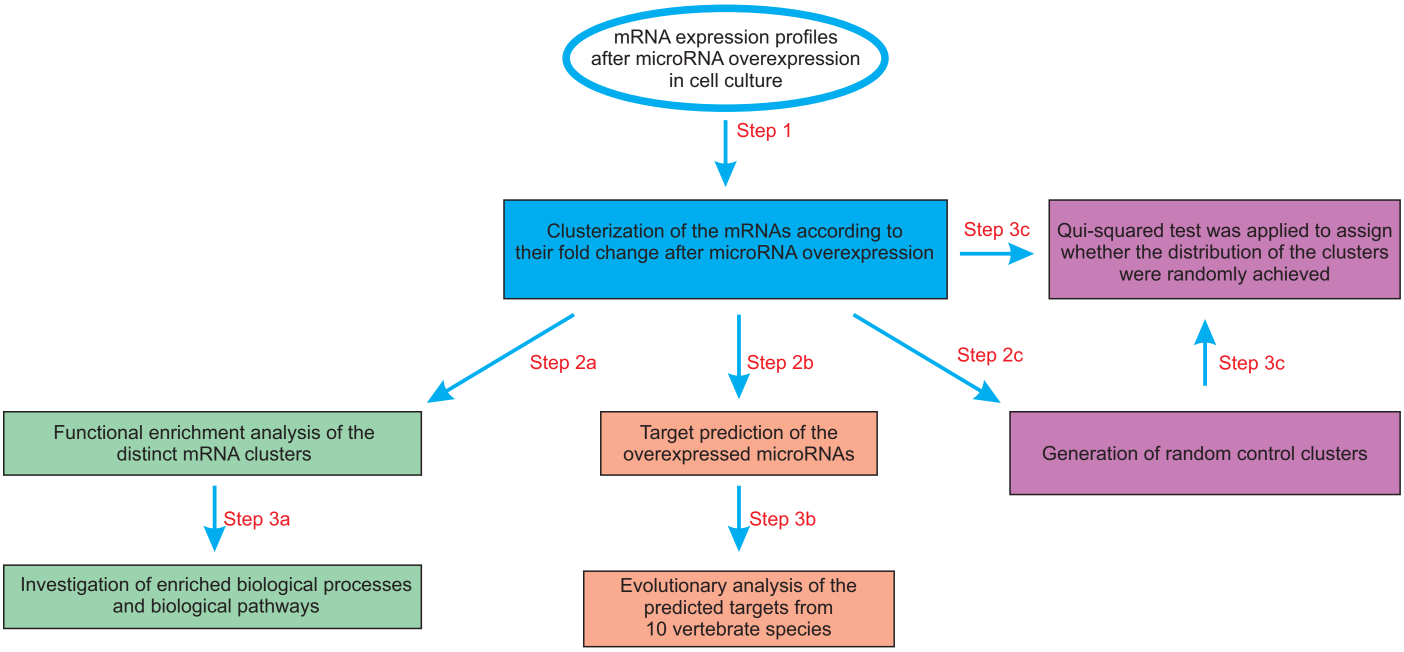

**Supplementary Figure S2.** Number of clusters identified for the 10 miRNAs analyzed. For each miRNA, mRNAs with similar fold changes (log2) were clustered together using the software Weka (v3.8; Frank et al., 2016) by the k-means method. Nine clusters were tested (from two to ten) and the best cluster number for each array were identified with the elbow curve method, using a python algorithm developed by Sapota et al. (2011).

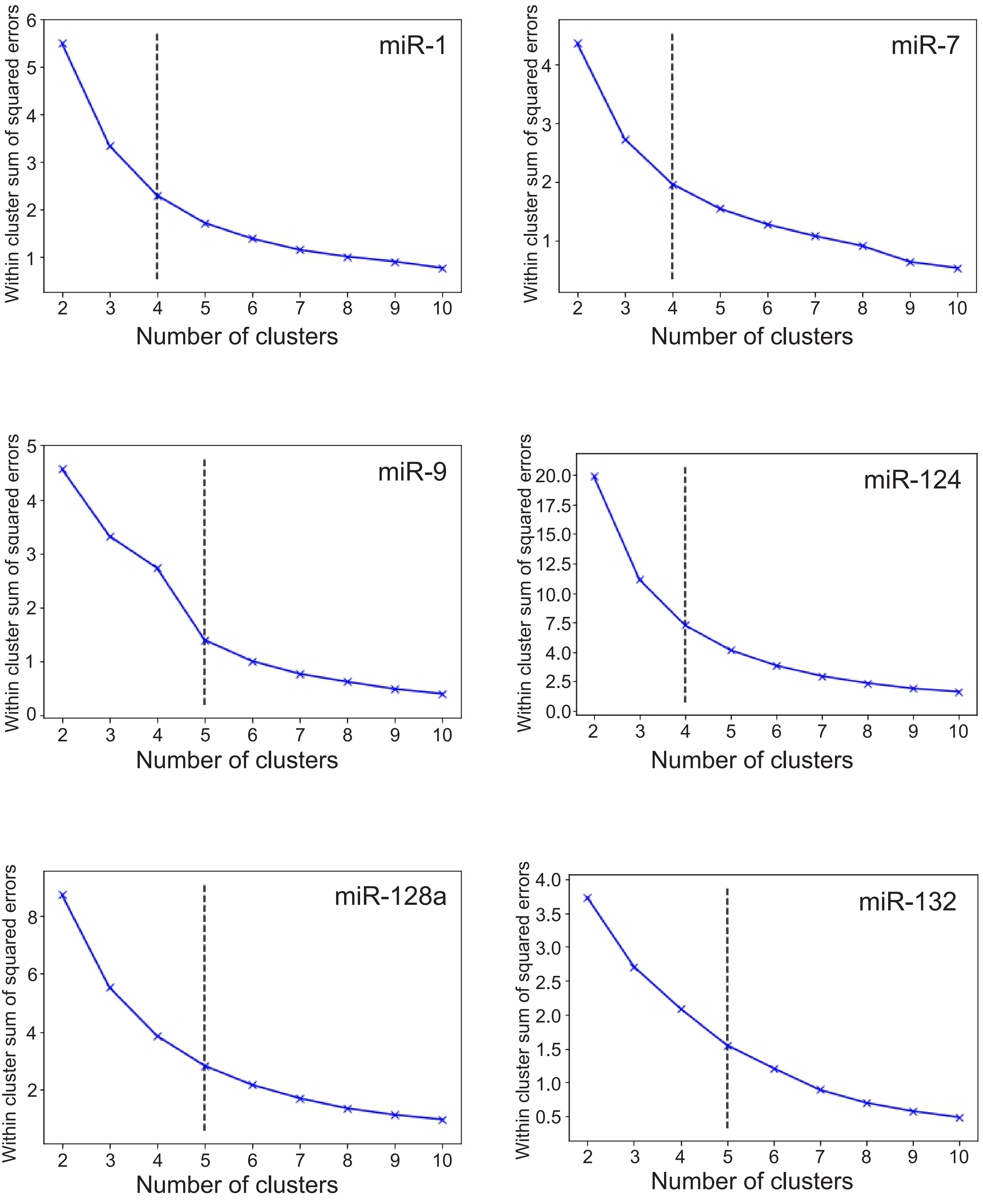

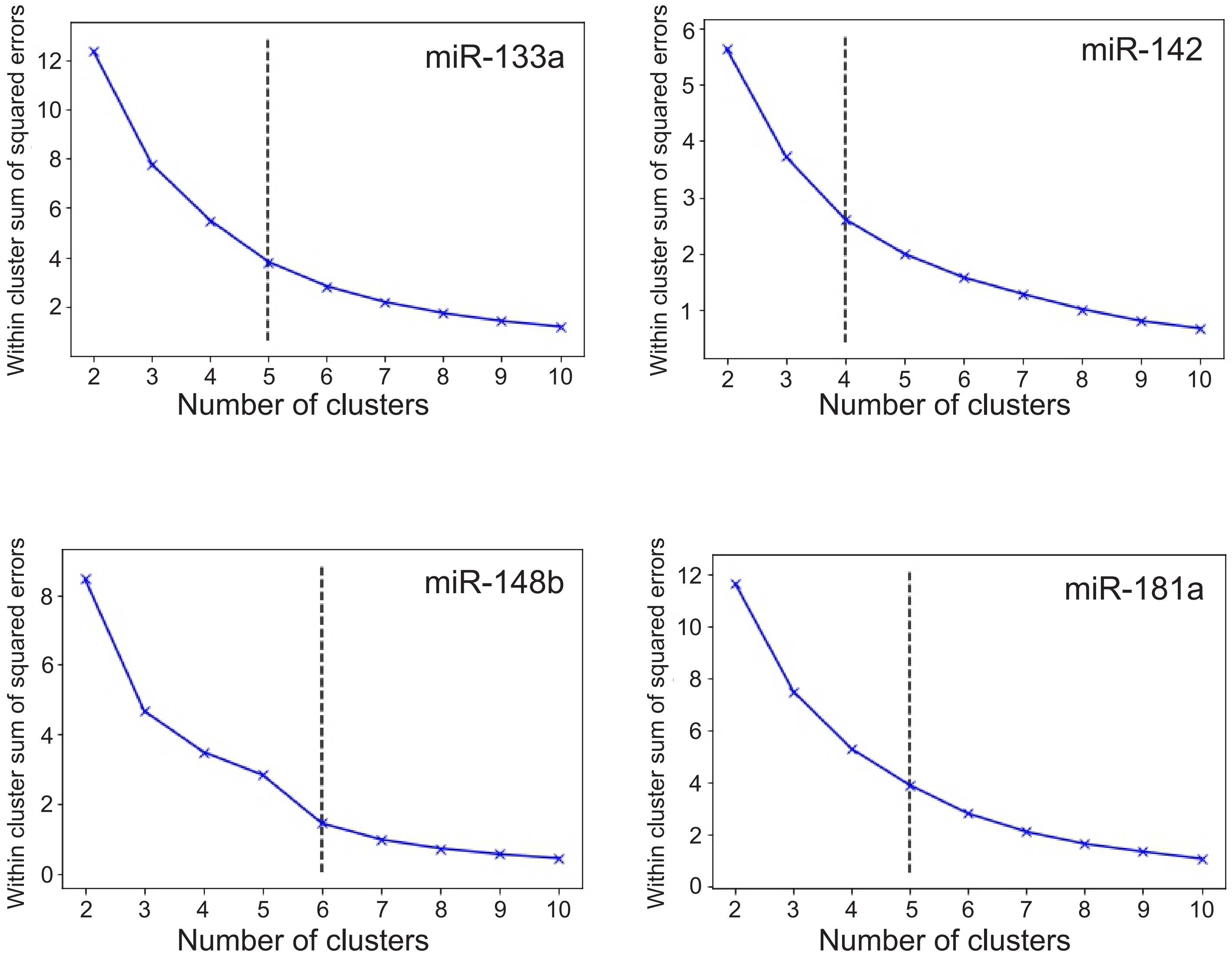

miR-133a
miR-142
miR-148b
miR-181a
Within cluster sum of squared errors
Number of clusters

**Supplementary Figure S3.** Correlation between the number of enriched terms and perturbed targets of the random sets of the 10 miRNAs analyzed. The average number of terms of the three random sets were used. Clusters are ordered by the number of gene members.

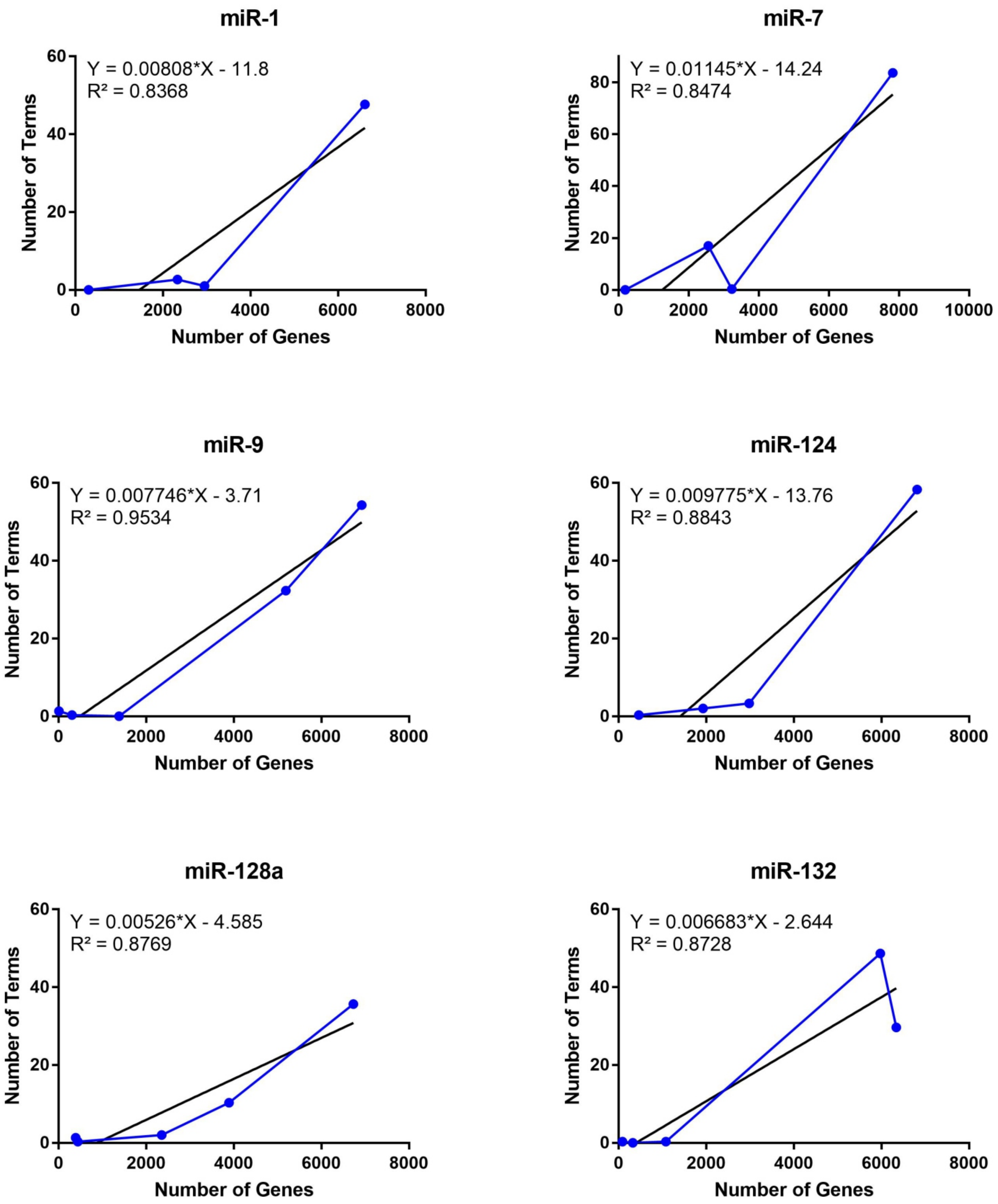

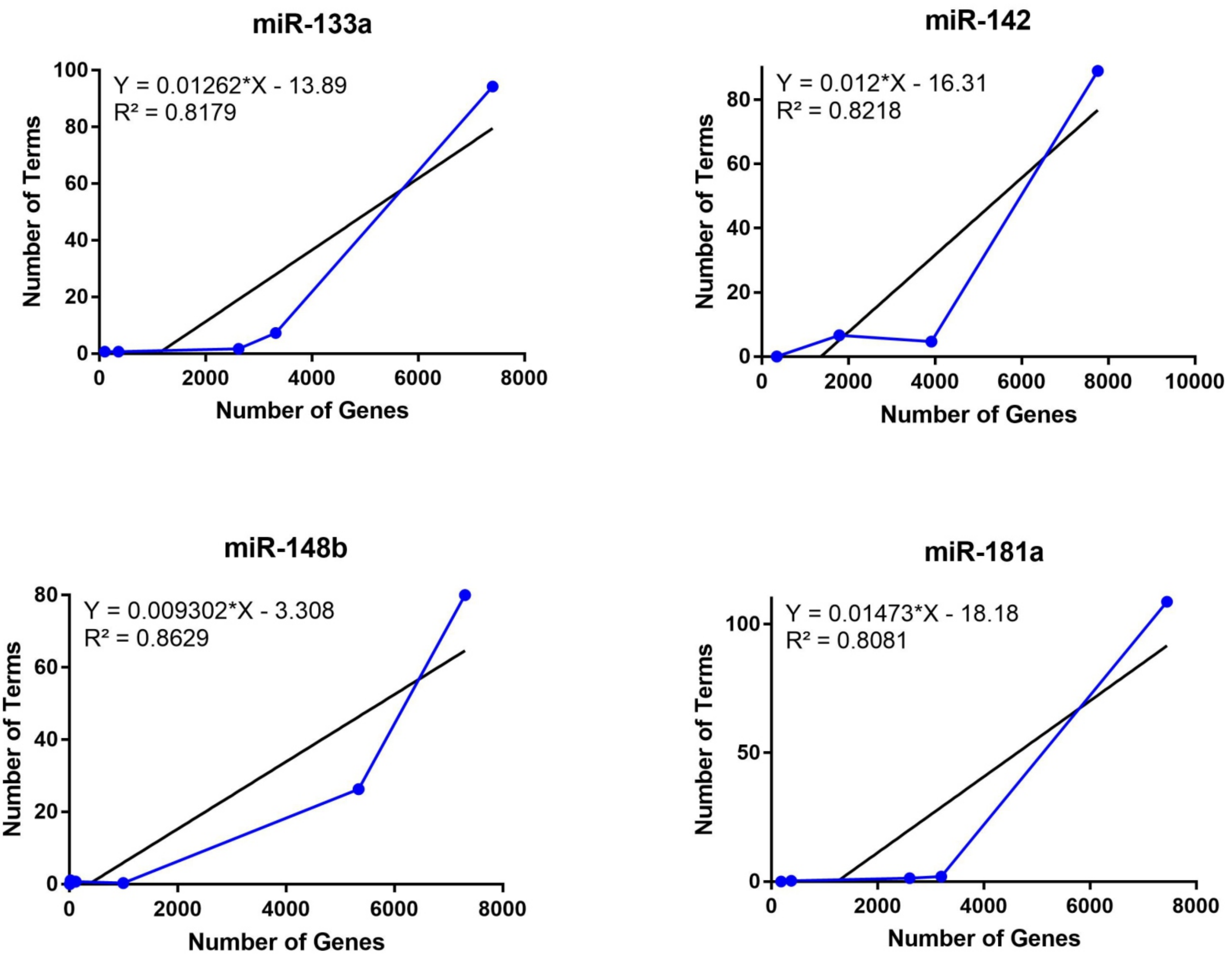
miR-133a
Y = 0.01262*X - 13.89
R² = 0.8179
Number of Terms
Number of Genes
miR-142
Y = 0.012*X - 16.31
R² = 0.8218
Number of Terms
Number of Genes
miR-148b
Y = 0.009302*X - 3.308
R² = 0.8629
Number of Terms
Number of Genes
miR-181a
Y = 0.01473*X - 18.18
R² = 0.8081
Number of Terms
Number of Genes

**Supplementary Figure S4.** Semantic similarity of the enriched GO terms of mRNA clusters after transfection of the 10 miRNAs. Each term enriched in the same miRNA cluster is identified by the same color. Enriched GO terms were identified by ToppCluster and summarized by REVIGO to eliminate redundant terms. The numbers within the brackets represent the minimum and maximum fold change values of each cluster. Terms with different colors in a cluster indicate that the same term is also present in the cluster of that color. Grey edges connect highly similar terms, with edge width indicating similarity degree. The name of the enriched terms of each cluster identified by ToppCluster can be found at Supplementary Table S3.

# miR-1

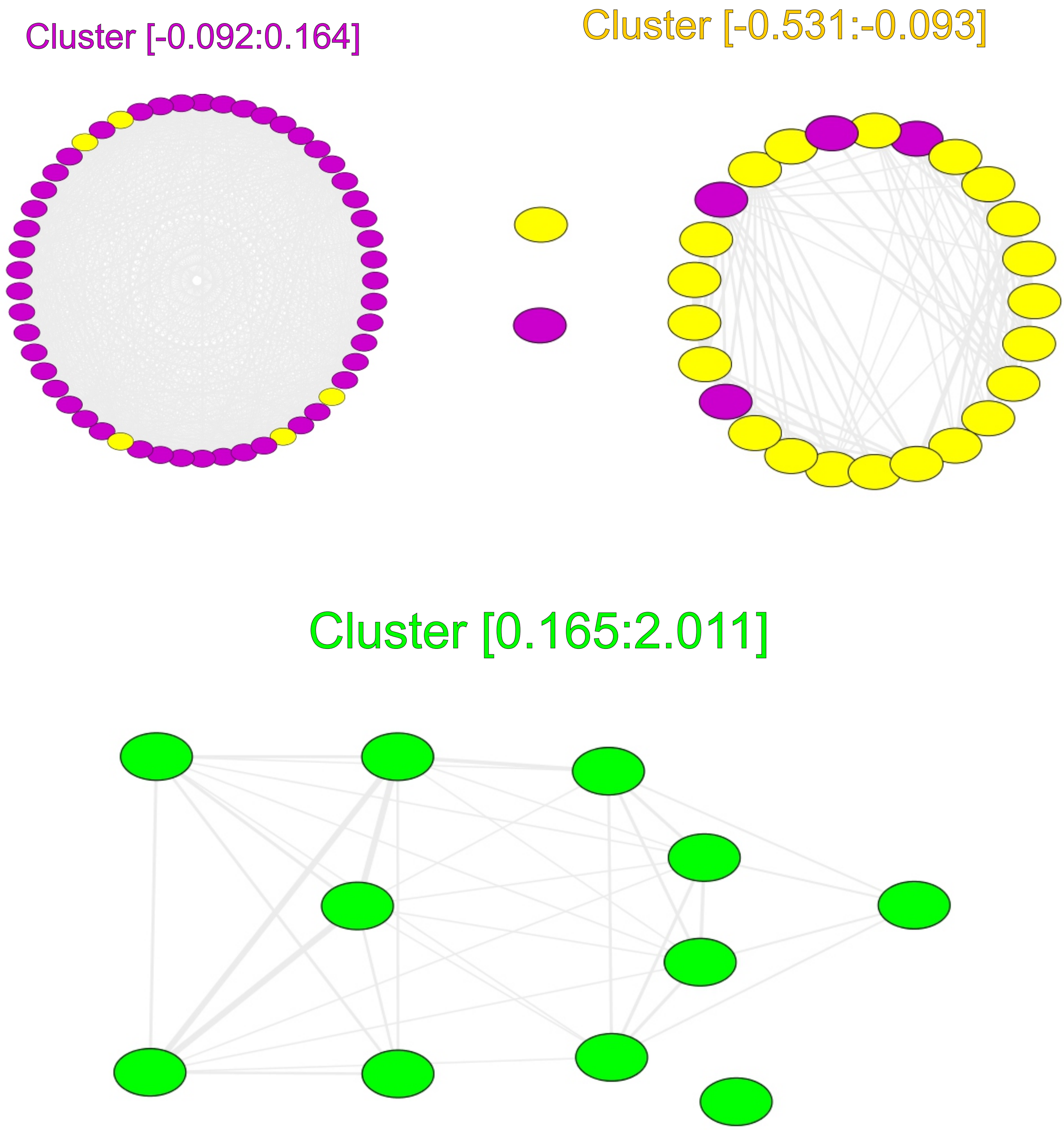

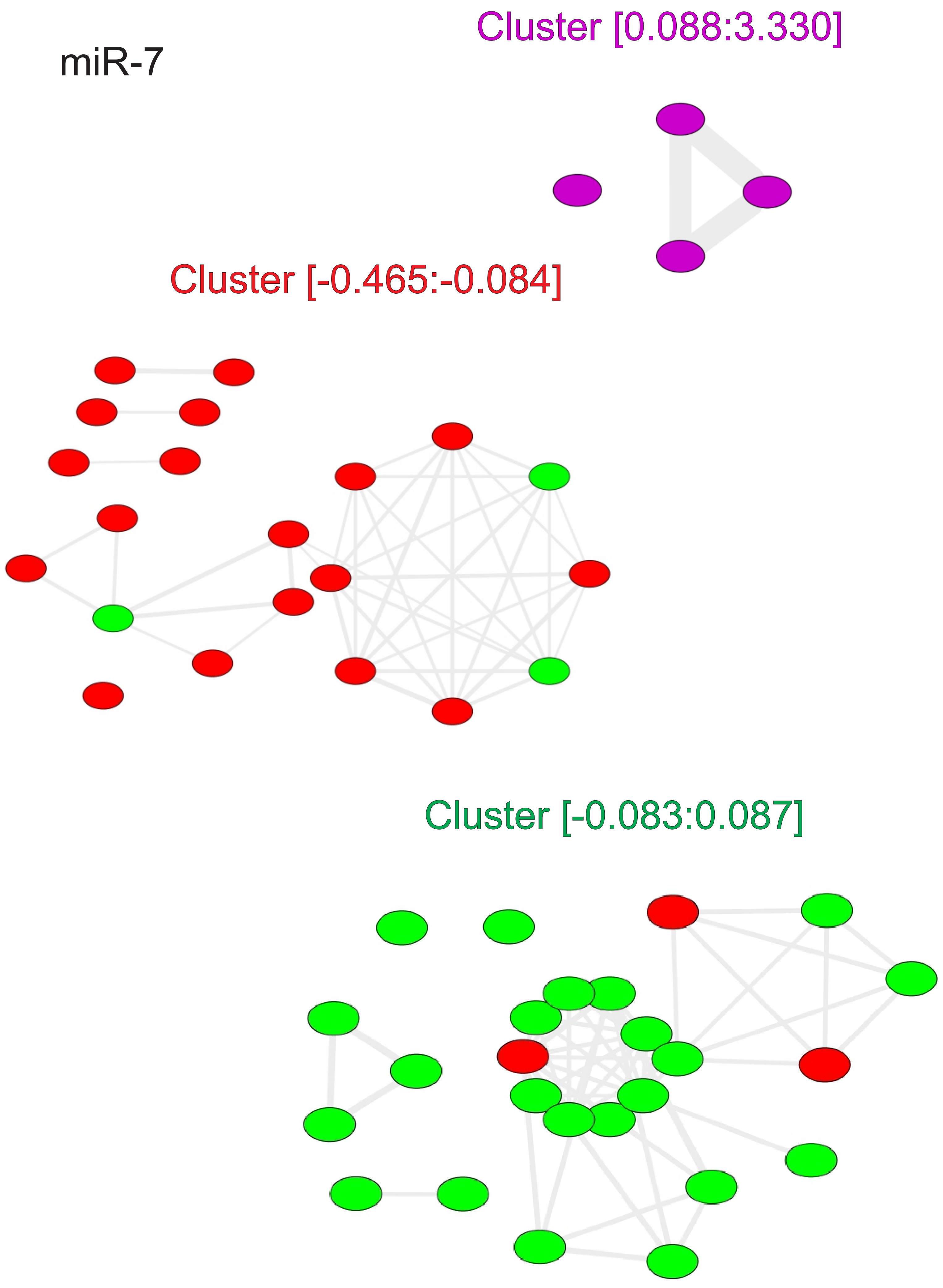
miR-7
Cluster [0.088:3.330]
Cluster [-0.465:-0.084]
Cluster [-0.083:0.087]

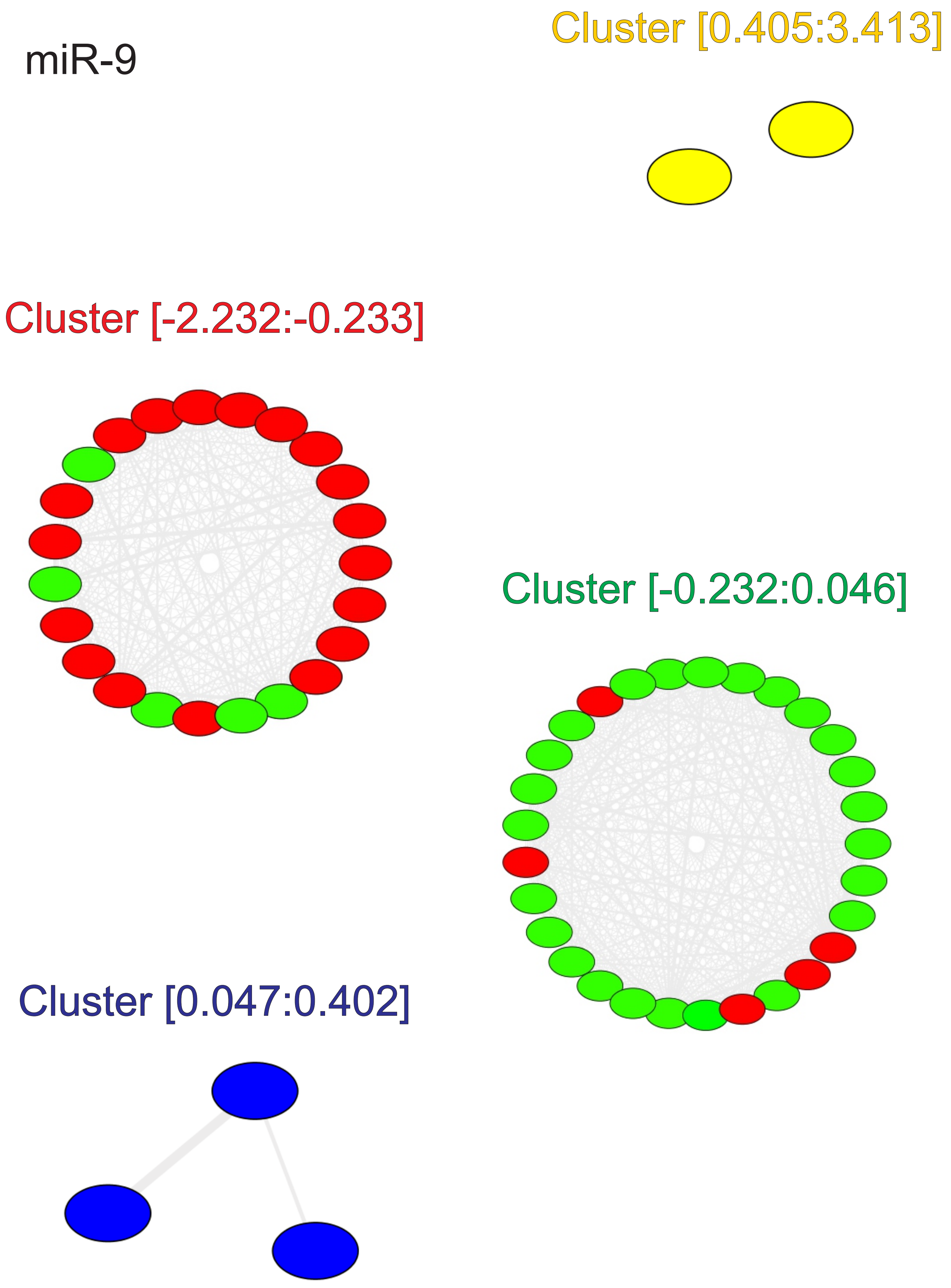
miR-9
Cluster [0.405:3.413]
Cluster [-2.232:-0.233]
Cluster [-0.232:0.046]
Cluster [0.047:0.402]

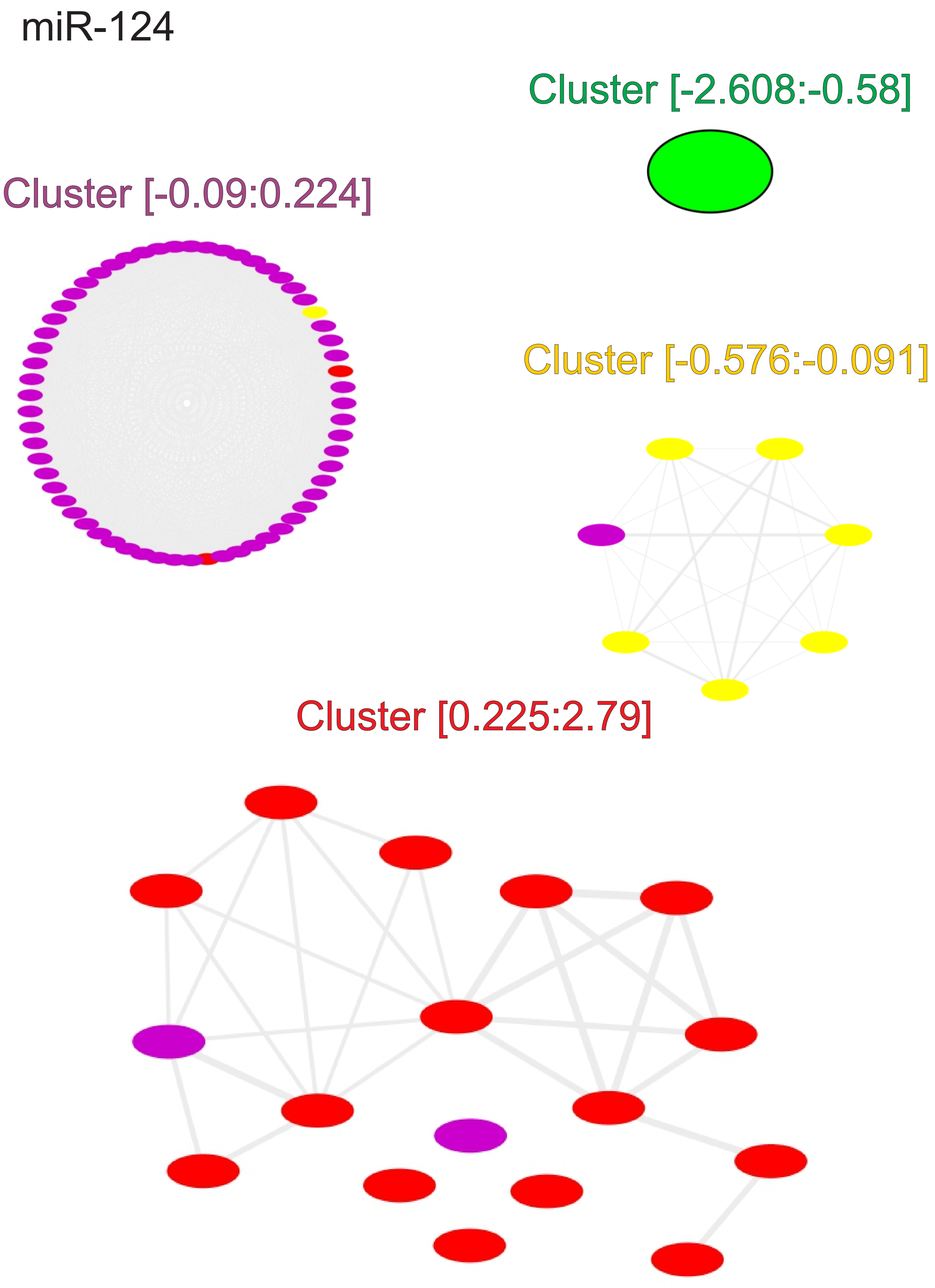

miR-124
Cluster [-2.608:-0.58]
Cluster [-0.09:0.224]
Cluster [-0.576:-0.091]
Cluster [0.225:2.79]

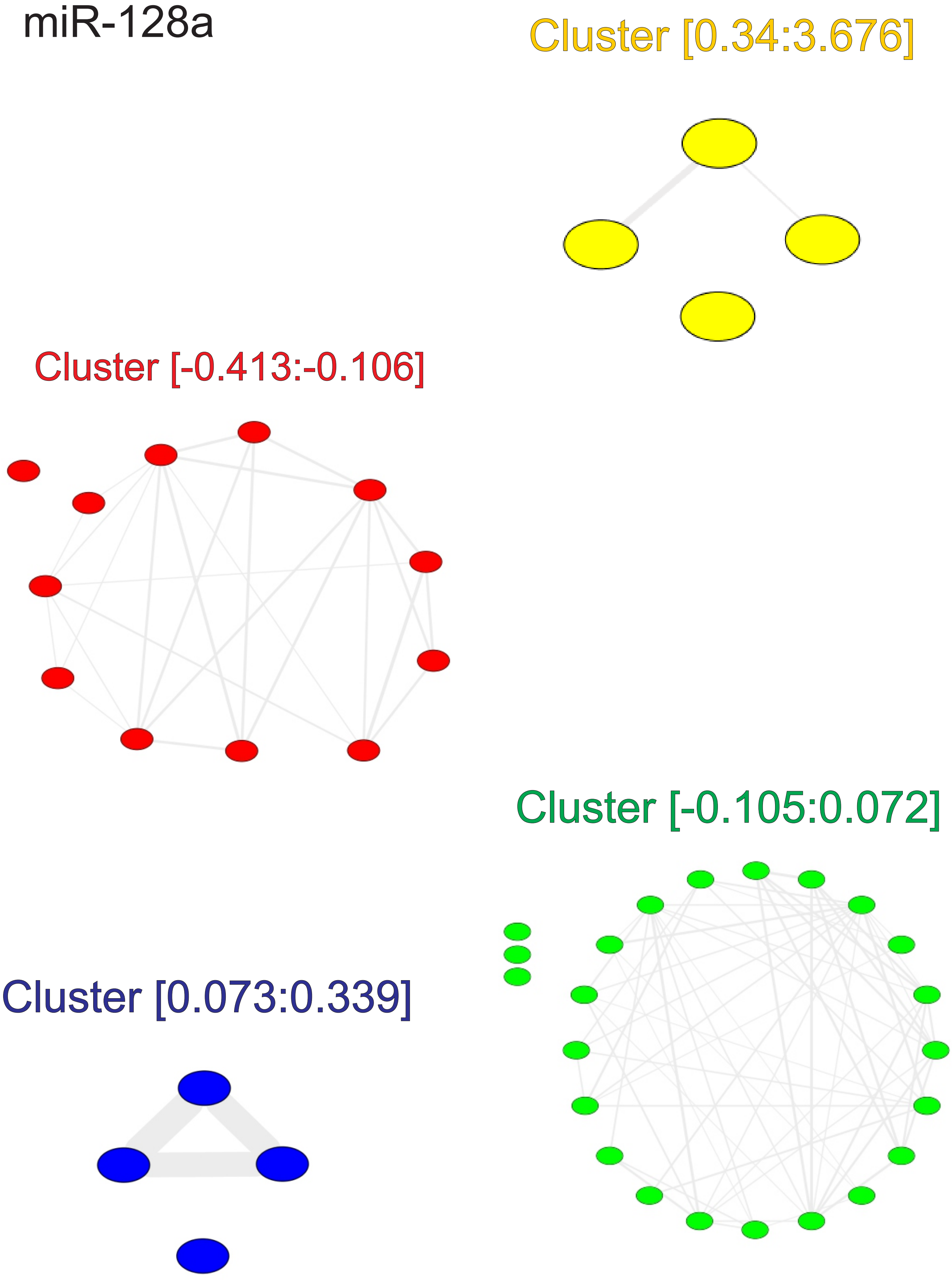
miR-128a
Cluster [0.34:3.676]
Cluster [-0.413:-0.106]
Cluster [-0.105:0.072]
Cluster [0.073:0.339]

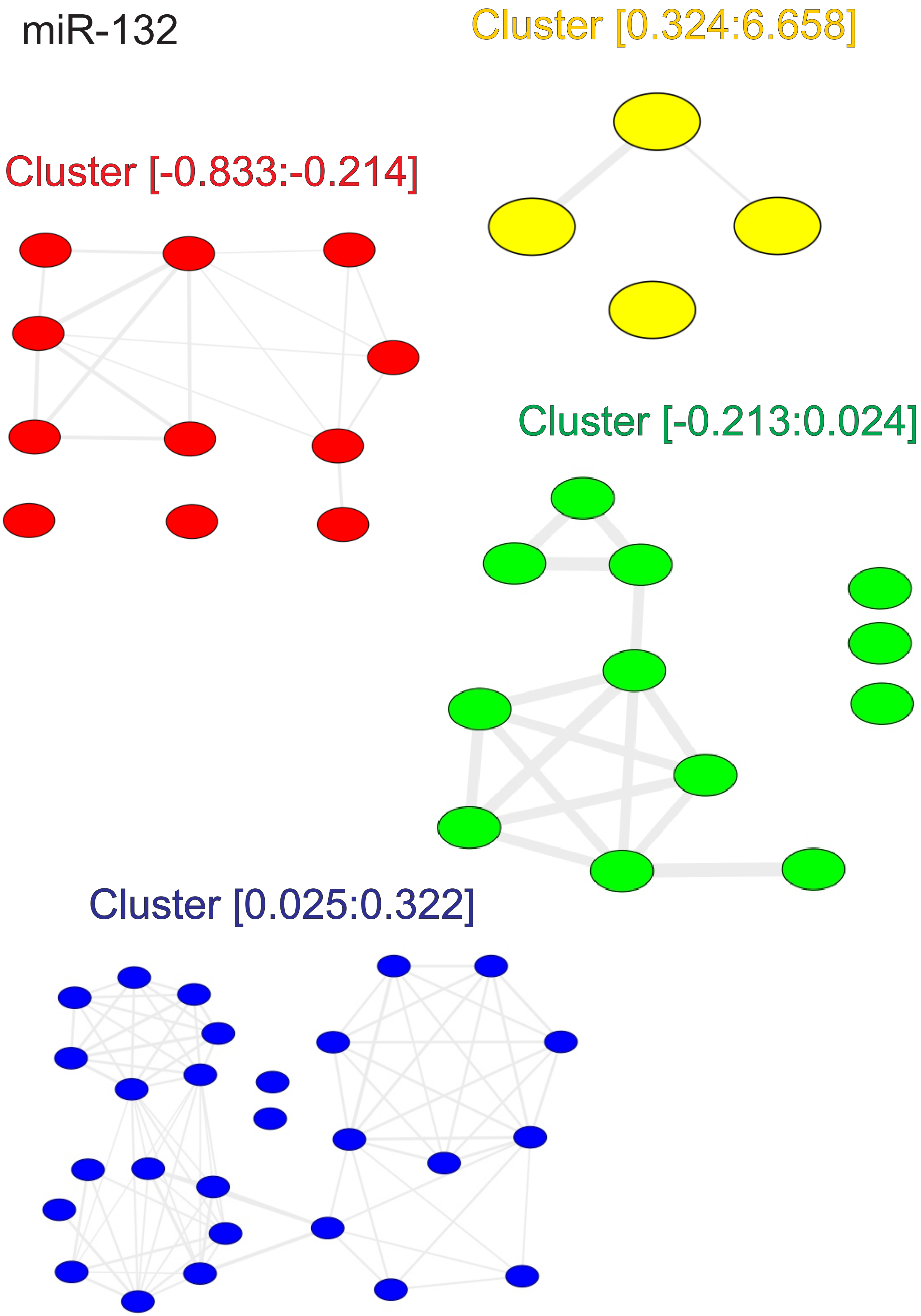
miR-132
Cluster [0.324:6.658]
Cluster [-0.833:-0.214]
Cluster [-0.213:0.024]
Cluster [0.025:0.322]

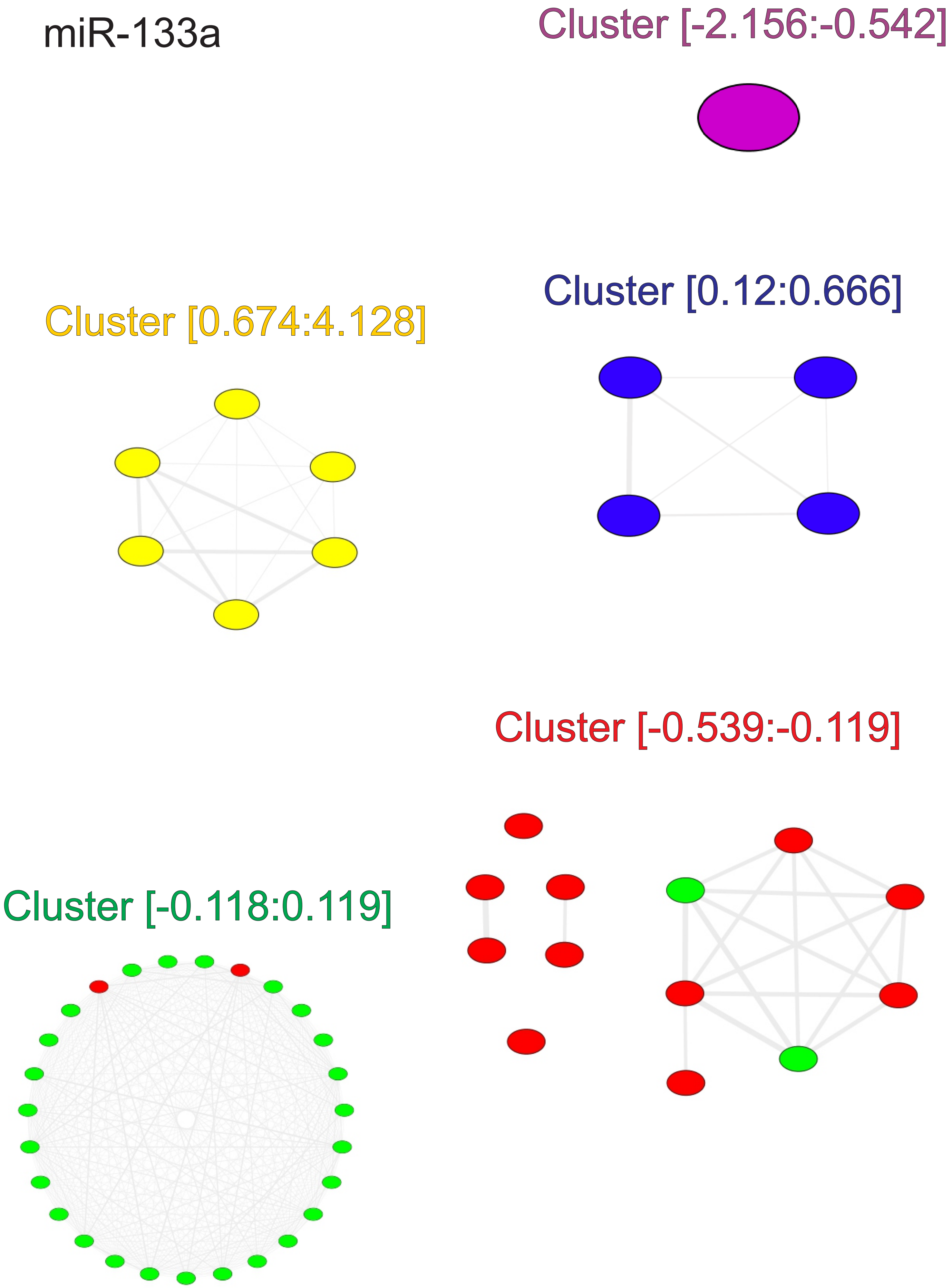
miR-133a
Cluster [-2.156:-0.542]
Cluster [0.674:4.128]
Cluster [0.12:0.666]
Cluster [-0.539:-0.119]
Cluster [-0.118:0.119]

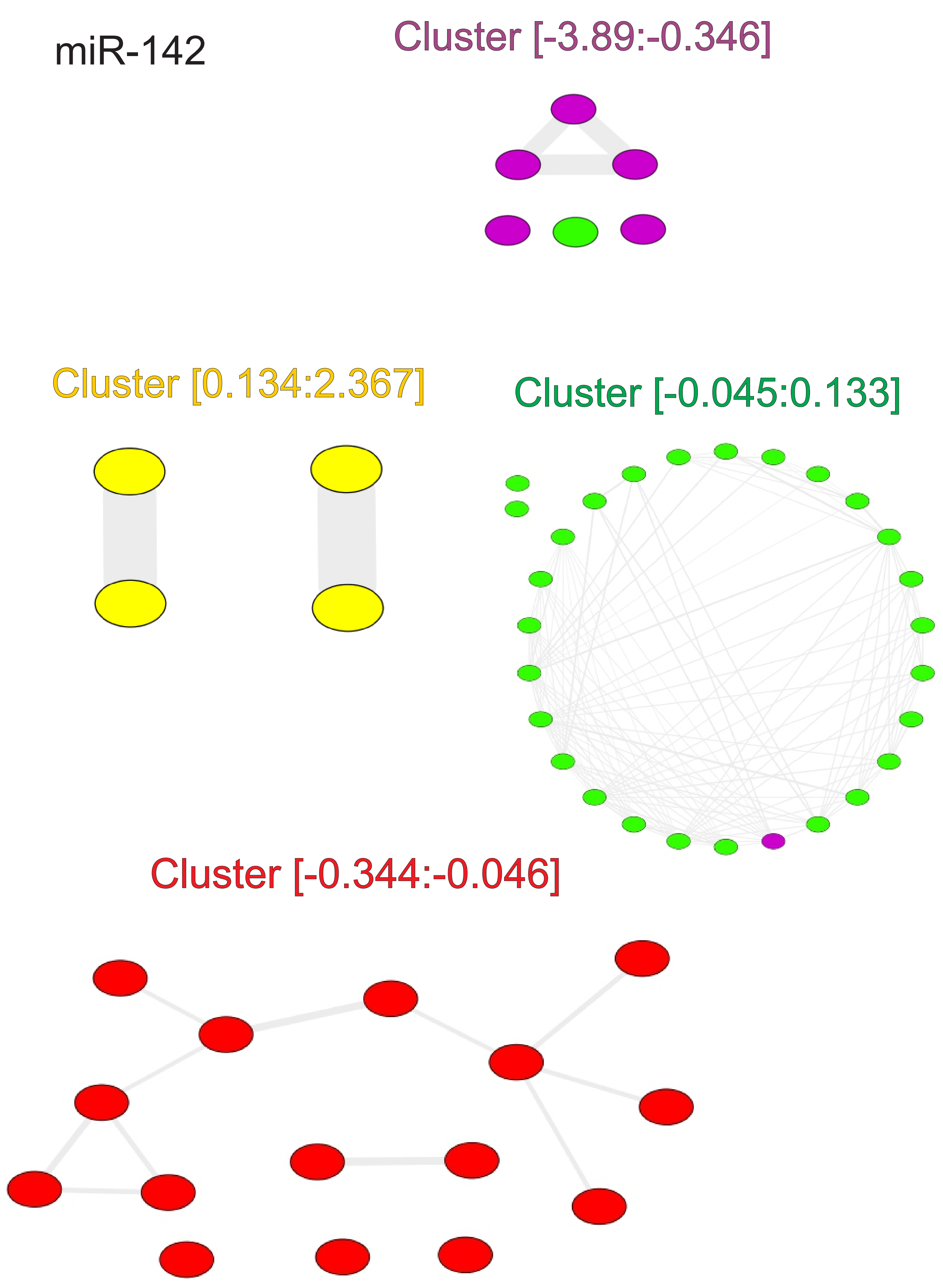
miR-142
Cluster [-3.89:-0.346]
Cluster [0.134:2.367]
Cluster [-0.045:0.133]
Cluster [-0.344:-0.046]

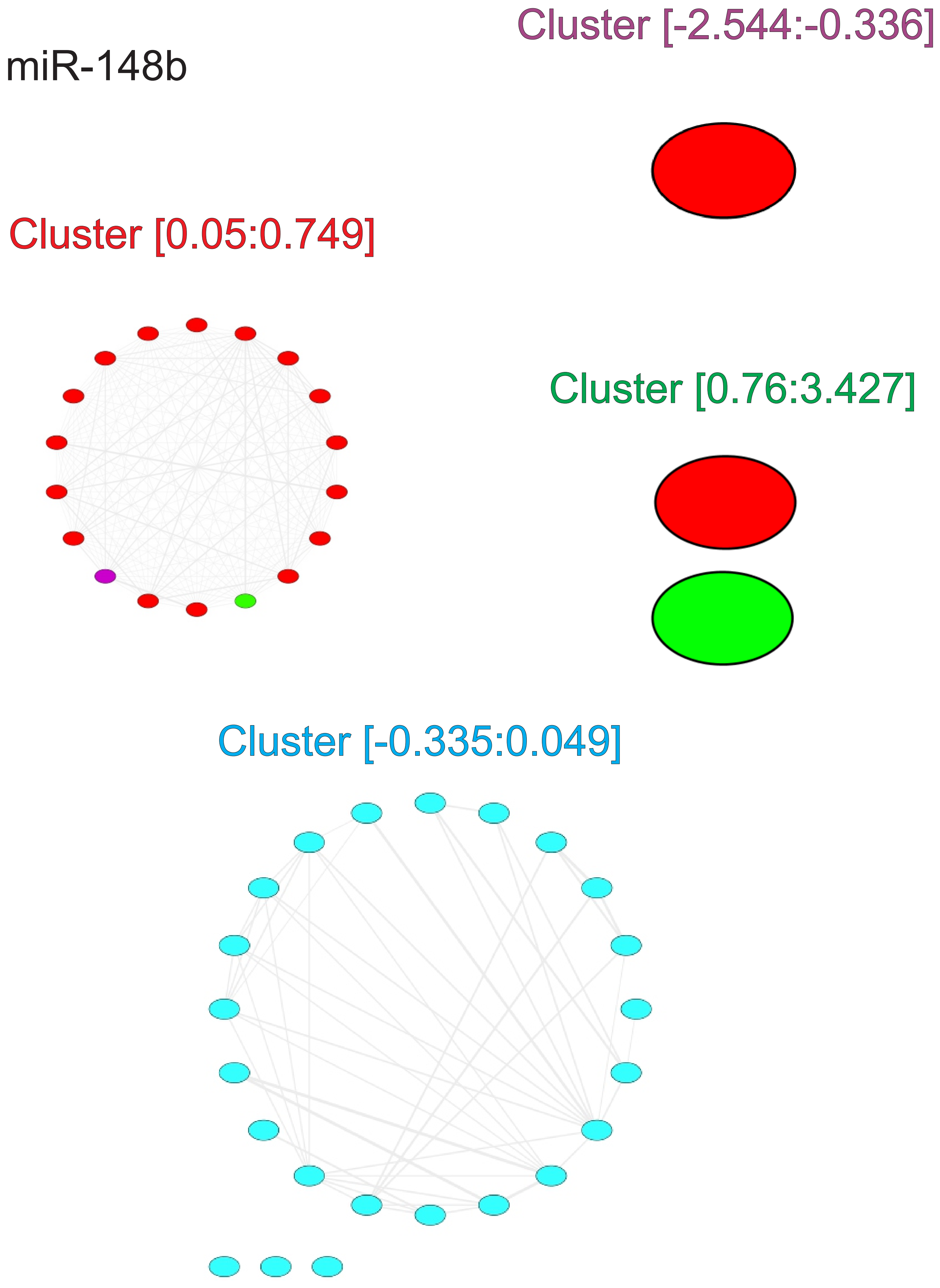
miR-148b
Cluster [-2.544:-0.336]
Cluster [0.05:0.749]
Cluster [0.76:3.427]
Cluster [-0.335:0.049]

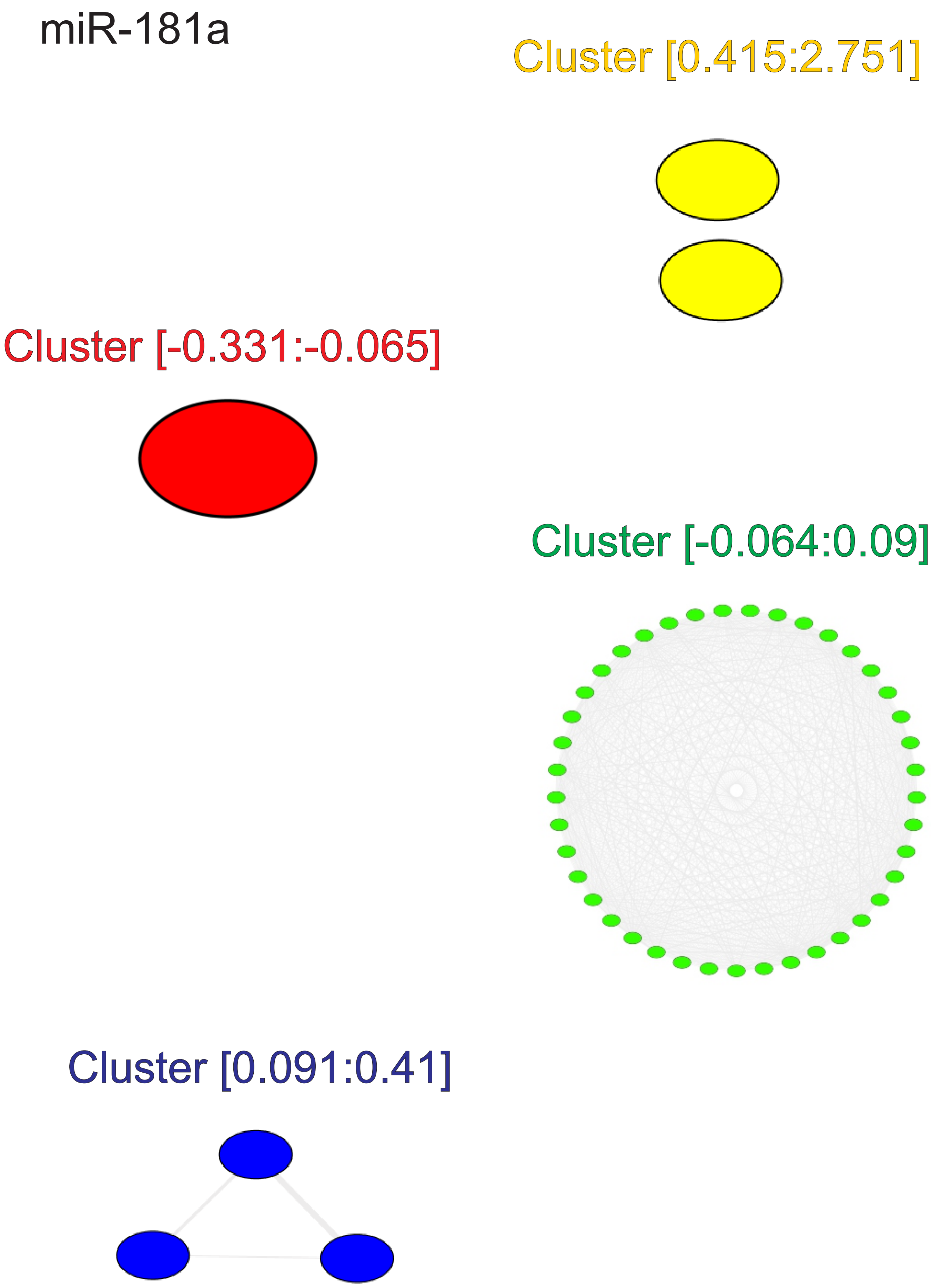
miR-181a
Cluster [0.415:2.751]
Cluster [-0.331:-0.065]
Cluster [-0.064:0.09]
Cluster [0.091:0.41]

**Supplementary Figure S5.** Correlation between mRNA fold change and the number predicted targets of the 10 miRNAs.

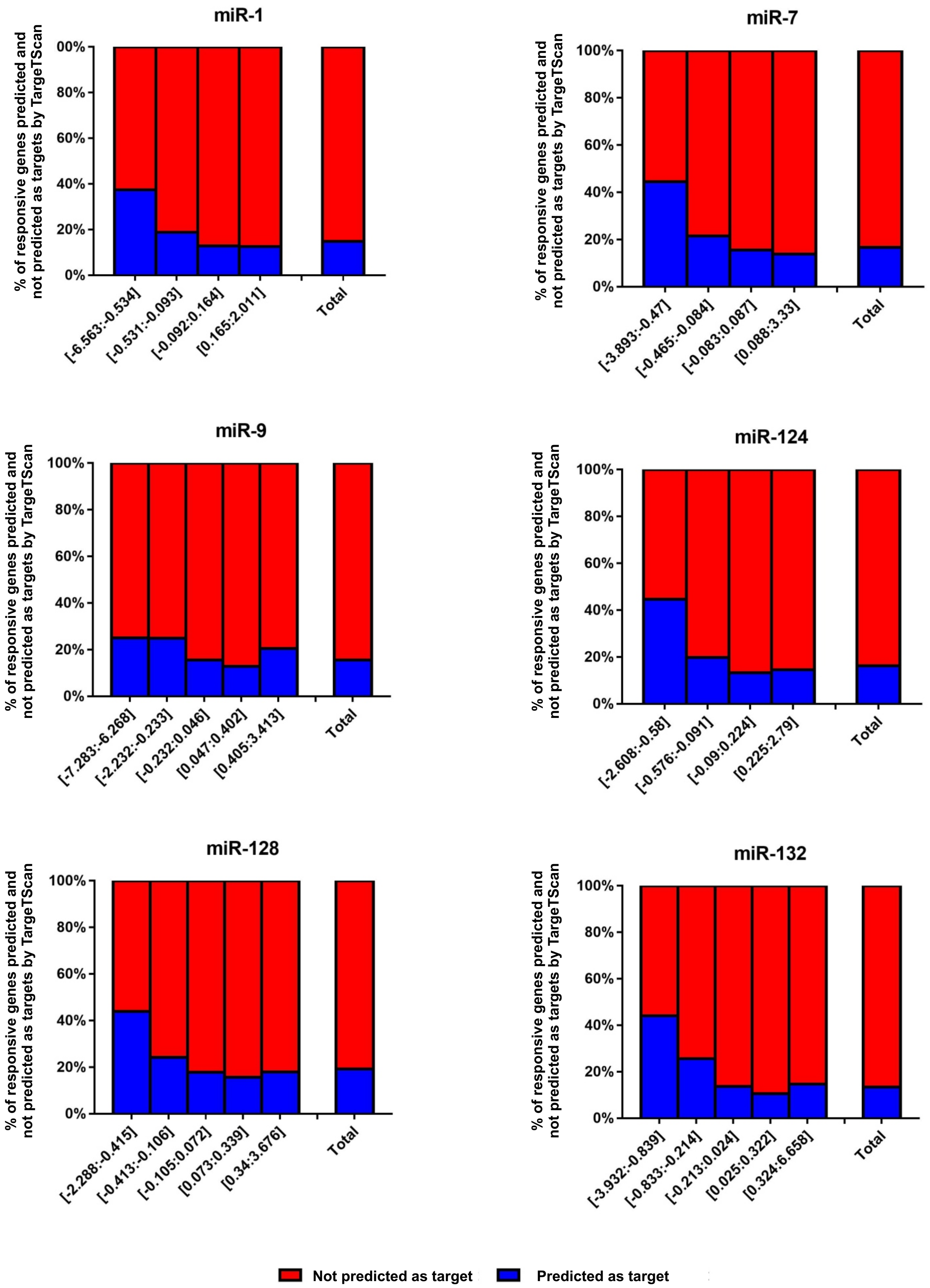

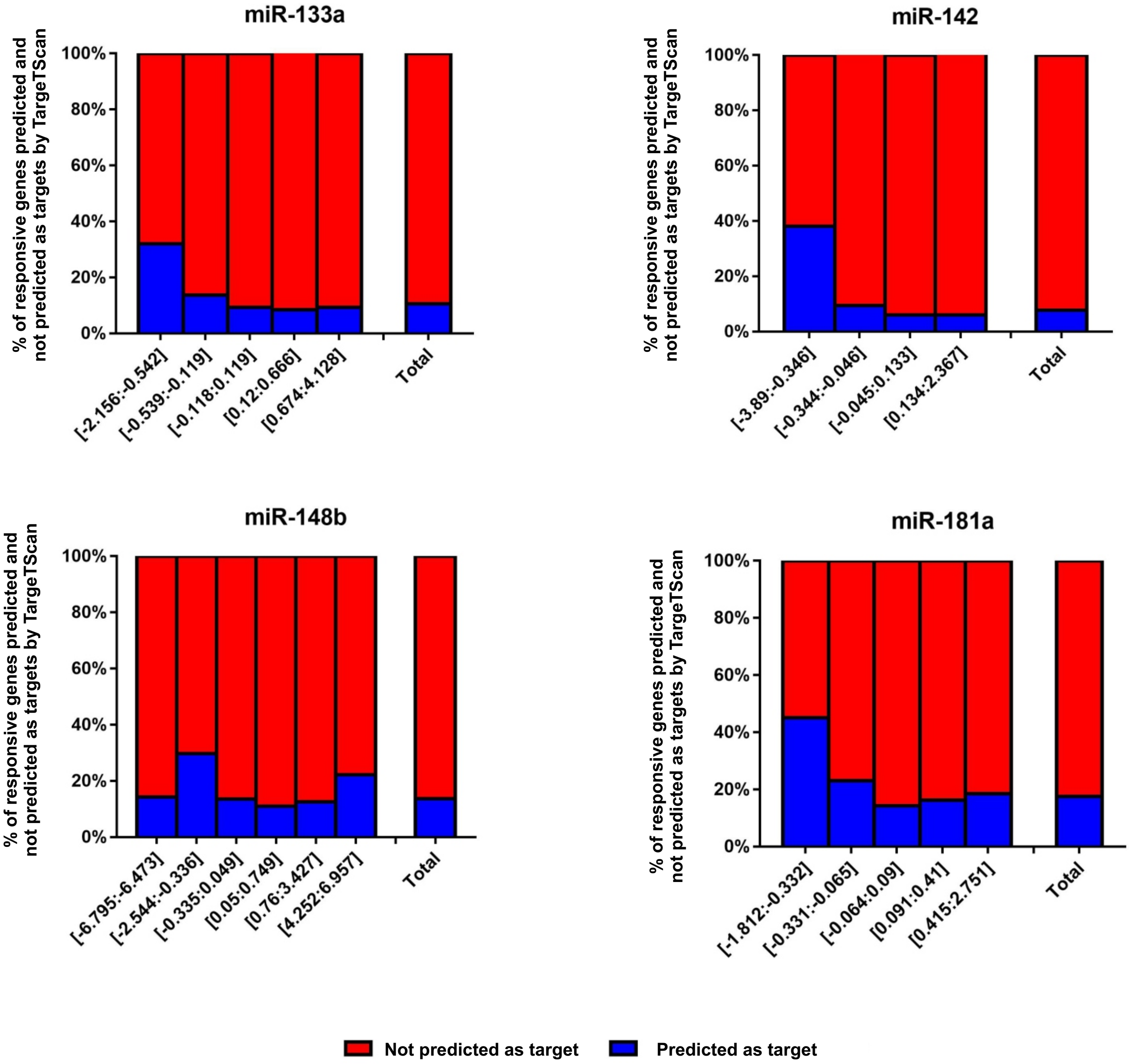
miR-133a
% of responsive genes predicted and not predicted as targets by TargeTScan
100%
80%
60%
40%
20%
0%
[-2.156:-0.542]
[-0.539:-0.119]
[-0.118:0.119]
[0.12:0.666]
[0.674:4.128]
Total
miR-142
[-3.89:-0.346]
[-0.344:-0.046]
[-0.045:0.133]
[0.134:2.367]
Total
miR-148b
[-6.795:-6.473]
[-2.544:-0.336]
[-0.335:0.049]
[0.05:0.749]
[0.76:3.427]
[4.252:6.957]
Total
miR-181a
[-1.812:-0.332]
[-0.331:-0.065]
[-0.064:0.09]
[0.091:0.41]
[0.415:2.751]
Total
Not predicted as target
Predicted as target

**Supplementary Figure S6.** Correlation between mRNA fold change and Context++Score. To perform this correlation, for each miRNA we separated the targets predicted by TargetScan in five groups according to its Context++Score ([0.1:0.2[; [0.2:0.3[; [0.3:0.4[; [0.4:0.5[ and [0.5:0.6[). Then we calculated the average fold change of the predicted targets of each group. Finally, for each group, we calculated the average fold change of the ten miRNAs.

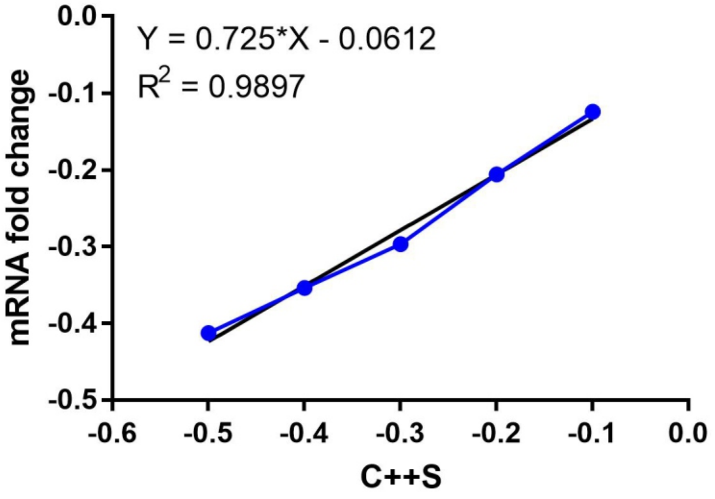

**Supplementary Figure S7.** Heatmaps of Context++Scores of the predicted targets of the ten miRNAs analyzed among ten vertebrate species. Negative Context++Score values are represented in green, positive values are represented in red, and near-zero values are represented in black. Lower Context++Score values represent stronger miRNA regulation levels.

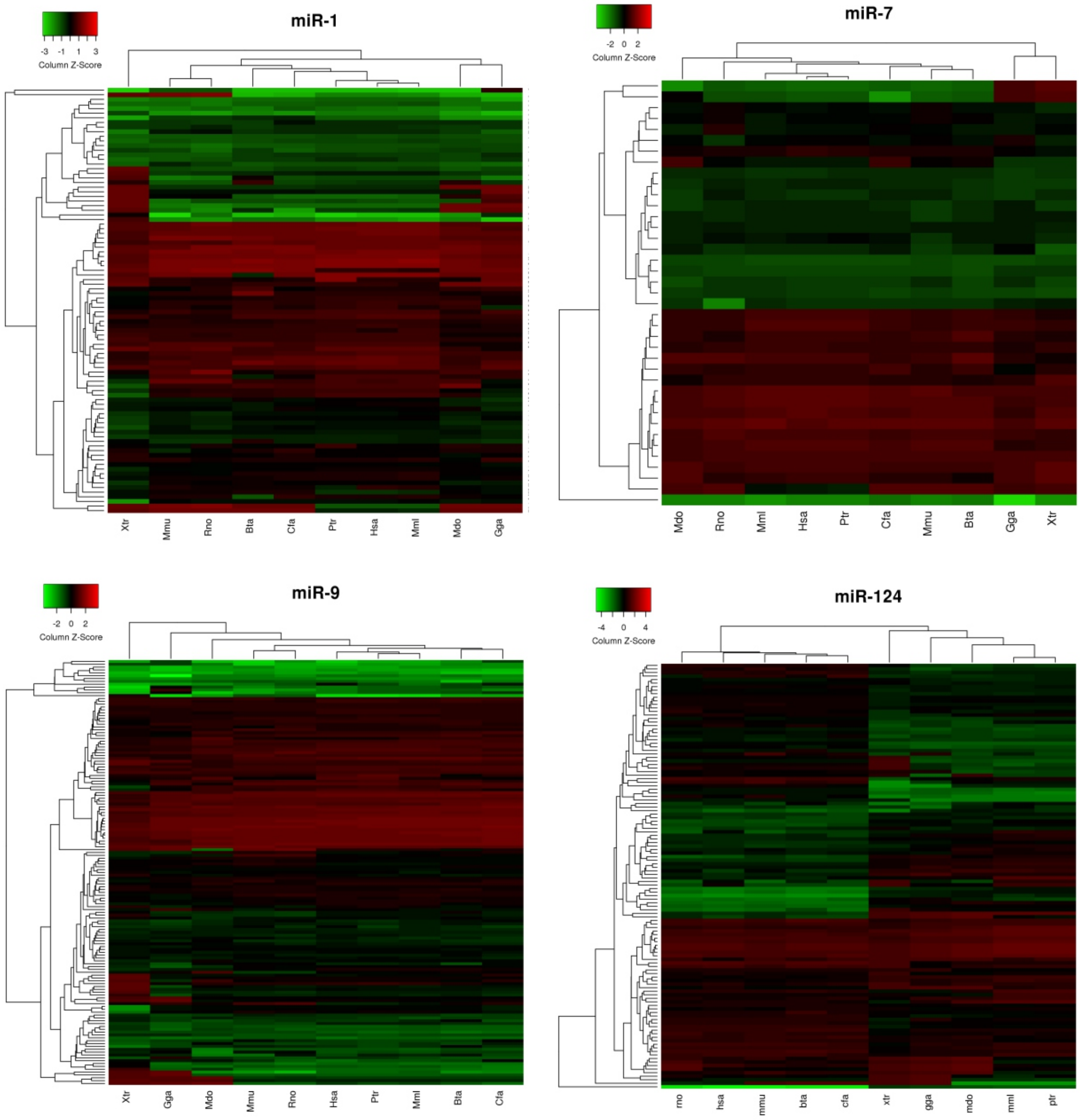

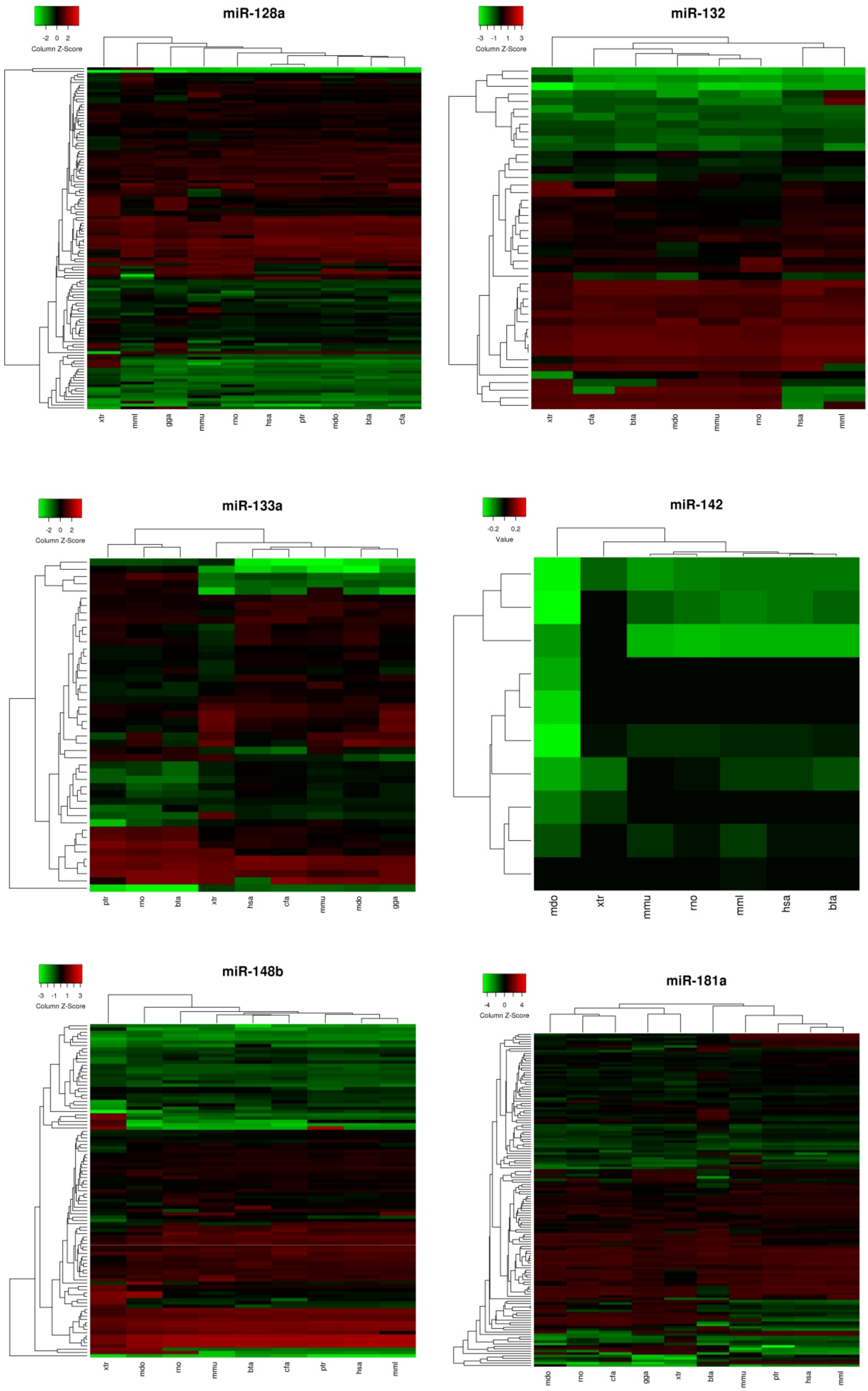
miR-128a
-2 0 2
Column Z-Score
xtr mml gga mmu rno hsa ptr mdo bta cfa
miR-132
-3 -1 1 3
Column Z-Score
xtr cfa bta mdo mmu rno hsa mml
miR-133a
-2 0 2
Column Z-Score
ptr rno bta xtr hsa cfa mmu mdo gga
miR-142
-0.2 0.2
Value
mdo xtr mmu rno mml hsa bta
miR-148b
-3 -1 1 3
Column Z-Score
xtr mdo rno mmu bta cfa ptr hsa mml
miR-181a
-4 0 4
Column Z-Score
mdo rno cfa gga xtr bta mmu ptr hsa mml
